# Functionalized Indolizines as Potential Anticancer Agents: Synthetic, Biological and In Silico Investigations

**DOI:** 10.3390/ijms26178368

**Published:** 2025-08-28

**Authors:** Roxana Ciorteanu, Catalina Ionica Ciobanu, Narcis Cibotariu, Sergiu Shova, Vasilichia Antoci, Ionel I. Mangalagiu, Ramona Danac

**Affiliations:** 1 Institute of Interdisciplinary Research—RECENT AIR Center, Alexandru Ioan Cuza University of Iasi, 11 Carol I, 700506 Iasi, Romania; roxana.ciorteanu@uaic.ro; 2 Faculty of Chemistry, Alexandru Ioan Cuza University of Iasi, 11 Carol I, 700506 Iasi, Romania; vasilichia.antoci@uaic.ro (V.A.); ionelm@uaic.ro (I.I.M.); 3 Institute of Interdisciplinary Research—CERNESIM Centre, Alexandru Ioan Cuza University of Iasi, 11 Carol I, 700506 Iasi, Romania; catalina.ciobanu@uaic.ro; 4 Centre of Advanced Research in Bionanoconjugates and Biopolymers, “Petru Poni” Institute of Macromolecular Chemistry of Romanian Academy, 41A Grigore Ghica Voda Alley, 700487 Iasi, Romania; cibotariu.narcis@icmpp.ro; 5 Department of Inorganic Polymers, “Petru Poni” Institute of Macromolecular Chemistry of Romanian Academy, 41A Grigore Ghica Voda Alley, 700487 Iasi, Romania; shova@icmpp.ro

**Keywords:** substituted indolizines, 3+2 cycloaddition, anticancer, molecular docking, ADMET

## Abstract

Three new series of indolizines (**5a**–**f**, **6a**–**f** and **7a**–**g**), functionalized with bromine or ethyl ester substituents on the pyridine ring, were designed and synthesized as promising anticancer agents. The synthesis of indolizine derivatives was carried out using the 1,3-dipolar cycloaddition of pyridinium *N*-ylides to ethyl propiolate as a key step. Spectral characterization (using NMR, FT-IR, HRMS and X-ray diffraction) showed that two types of cycloadducts **5a**–**f** and **6a**–**f** were obtained when the ylides generated by the 3-bromopyridinium salts were used as 1,3-dipoles in Huisgen cycloaddition reactions to ethyl propiolate. The anticancer effect of selected compounds was in vitro assessed against the National Cancer Institute (NCI) panel of 60 human tumor cells, at 10 μM concentration, with three compounds (**5c**, **6c** and **7g**) showing promising inhibitory activity on the growth of several cell lines including lung, brain, renal cancer and melanoma, as well as a cytotoxic effect against HOP-62 non-small cell lung cells (34% for compound **5c** and 15% for compound **7g**) and SNB-75 glioblastoma cells (15% for compound **5c** and 14% for derivative **7c**). Molecular docking revealed favorable binding affinities for **5c**, **6c** and **7g** (–9.22 to –9.88 kcal/mol) at the colchicine-binding site of tubulin with key interactions involving βASN-258, βALA-317, and βLYS-352 residues for **5c**, βASN-258 in case of **6c**, and αVAL-181 and βLYS-254 for derivative **7g**. According to the in silico ADMET analysis, the active compounds are predicted to exhibit good oral bioavailability, promising drug-like qualities and low toxicity risks.

## 1. Introduction

Heterocyclic compounds, particularly azaheterocycles, occupy a central role in the field of medicinal chemistry, due to their prevalence in bioactive molecules, including pharmaceuticals, natural products, and agrochemicals [1,2,3,4]. The heterocyclic scaffolds can mimic biological molecules and interact effectively with diverse biological targets, including enzymes, receptors, and nucleic acids. A substantial rise in drugs containing at least one nitrogen heterocycle—82%, up from 59% in previous decades—as well as an increase in the average number of nitrogen heterocycles per drug were observed at the examination of the structures and properties of 321 unique small-molecule drugs approved between January 2013 and December 2023 [3]. Moreover, the number of drugs incorporating fused heterocycles has grown significantly [3], as fusion often introduces conformational rigidity, which can improve binding to biological targets by aligning key functional groups in optimal orientations. Their structural versatility and functional diversity allow for extensive modification, enabling medicinal chemists to fine-tune their pharmacokinetic and pharmacodynamic properties [5,6,7,8]. The type of heterocyclic scaffold, its ring size, and substituents significantly influence the physicochemical properties, which have led to extensive research in developing novel heterocyclic frameworks to address a range of diseases [1,2,3,4,5,6,7,8,9].

Azaheterocyclic compounds exert their anticancer activity through diverse mechanisms, including the inhibition of protein kinases, modulation of microtubule dynamics, disruption of DNA replication via topoisomerase I/II inhibition or direct DNA intercalation, induction of oxidative stress and ferroptosis, and modulation of cell cycle regulators such as CDK1/Cyclin B [2,3,4,5,6,7,8,9].

Among them, indolizines, as fused bicyclic nitrogen systems, have gained attention due to their planar structure, extended conjugation, and capacity to form π-stacking and hydrogen bonding interactions with key biological targets. Indolizine-based compounds have demonstrated a range of biological activities, with notable efficacy in anticancer applications, including the inhibition of tubulin polymerization, EGFR signaling disruption and induction of apoptosis in various cancer cell lines [10,11,12,13,14,15,16,17,18,19].

**Figure 1:**
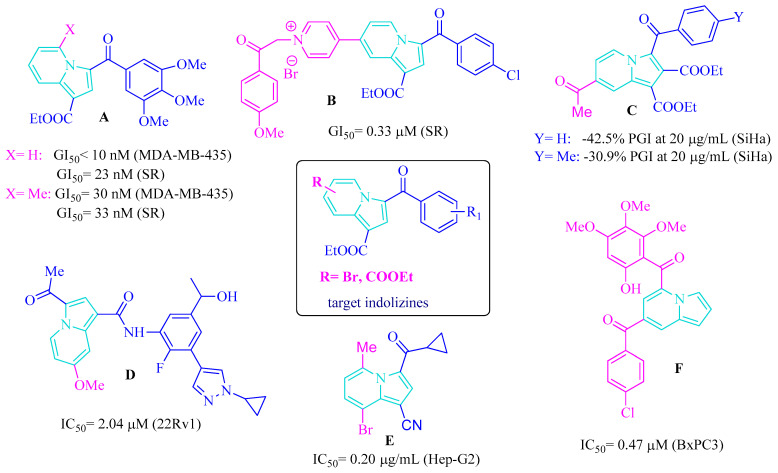
Structures of reported anticancer active indolizine substituted on the pyridine ring (**A**–**F**) [13,14,15,17,18,19] and the general structure of the target compounds.

Studies regarding structure–activity relationship (SAR) have identified some critical substitution patterns that significantly influence the anticancer potential of indolizine derivatives [10,11,12,13,14,15,16,17,18,19]. It is noteworthy that most reported anticancer indolizines bear substitutions on the pyrrole ring, whereas analogues modified on the pyridine ring were less investigated. Thus, we found indolizine bearing an unsubstituted pyridine ring or a simple methyl substituent at position 5 (compounds type **A**, Figure 1) that showed excellent antiproliferative properties against multiple cancer cell lines (with the highest potency against melanoma MDA-MB-435 cells and leukemia SR cells) and inhibition of tubulin polymerization [13,15]. Also, 5-methyl-8-bromoindolizine **E** (Figure 1) displayed anticancer activity against hepatocellular liver carcinoma Hep-G2 cell and significant EGRF kinase inhibitory activity [14]. Introduction of substituted benzoyl groups at positions 5 and 7 in indolizine derivative **F** (Figure 1) resulted in a strong inhibition of pancreatic BxPC3 cell viability [18]. Indolizine **B** bearing pyridinium-4-yl substituent at position 7 (Figure 1) displayed antiproliferative activity against several cancer cell lines [15], whereas substitution with an acetyl group at the same position yielded type **C** derivatives (Figure 1) with inhibitory properties on SiHa cervical cancer cells [17]. Indolizine **D** (Figure 1) carrying a methoxy substituent at position 7 also exhibited anticancer potency against prostate cancer cell lines 22Rv1 [19].

Although different substitution patterns on the pyridine ring of indolizine have been investigated for the anticancer activity, reports on halogenated indolizines are very limited. To date, only isolated examples such as 5-methyl-8-bromoindolizine **E** discussed above (Figure 1) or 5-chloro-2-(3-fluorophenyl)-3-(phenethylamino)indolizine-1- carbonitrile reported to inhibit migration of PC3 prostate cancer cell line [20] have been described.

The introduction of bromine has been widely utilized in medicinal chemistry to adjust the physicochemical and biological properties of drug candidates. Due to its size, polarizability, and ability to engage in halogen bonding, bromine can enhance binding affinity and target selectivity [21,22].

Also, the substitution with an ester group that can serve as a key hydrogen-bond acceptor was investigated, especially at the C-1 position, and has emerged as particularly advantageous. Thus, C1-ester-containing indolizines have consistently demonstrated enhanced cytotoxic potential compared to their acid or amide analogues [13].

Given the above data, the current study explores a series of new indolizine derivatives bearing bromine or carboxyethyl substituents on the pyridine ring of the indolizine—functionalization patterns that remain less investigated in the context of anticancer activity (Figure 1).

By investigating their synthesis, structure, anticancer effects, and physicochemical properties, this work aims to further elucidate the synergistic contributions of ester and bromine or two ester functionalities to the anticancer potential of indolizine-based pharmacophores. Through this investigation, we seek to expand the medicinal utility of the indolizine scaffold and provide a foundation for the rational design of next-generation anticancer agents. Also, we reported here an interesting reactivity of the ylides generated by 3-bromo-1-(2-oxo-2-phenylethyl)pyridin-1-ium bromides that generated two types of cycloadducts.

## 2. Results

### 2.1. Chemistry

**Scheme 1:**
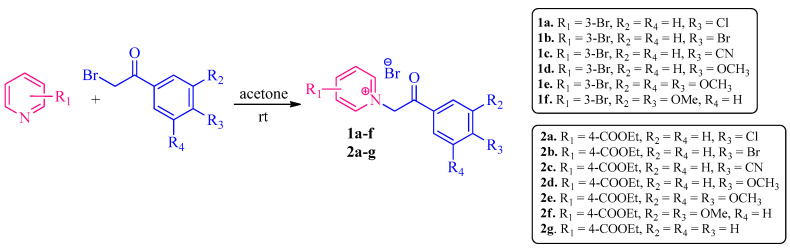
General scheme for the synthesis of quaternary salts **1a**–**f** and **2a**–**g**.

The synthetic approach to build the indolizine skeleton is based on a 3+2 cycloaddition of cycloimmonium ylides to ethyl propiolate, a strategy successfully applied in our group to synthesize various fused pyrrolo-heterocycles [14,15,16,23,24]. Therefore, the first step was the synthesis of monoquaternary pyridinium salts of 3-bromopyridine (compounds **1a**–**f**) and ethyl isonicotinate (compounds **2a**–**g**), respectively. The type **1** and **2** derivatives were prepared by the direct reaction of the corresponding substituted pyridine with 2-bromo-acetophenones that differ by the substituent at the phenyl ring, the reactions being carried out in acetone, at room temperature (rt) (Scheme 1).

Four of the type **2** monoquaternary salts (**2a**, **2b**, **2d** and **2g**) have been previously fully characterized in the literature [25], while salts **1a** and **1d** are the only ones from this series reported as intermediates in synthesis [26,27], but they were not spectrally characterized.

By treating salts **1** with triethylamine, the corresponding ylides have been in situ generated. It is known that cycloimmonium ylides can react as nucleophiles or as 1,3-dipole in the presence of electron-deficient dipolarophiles such as alkynes or alkenes to efficiently construct indolizine frameworks with high atom economy and relative structural diversity.

**Scheme 2:**
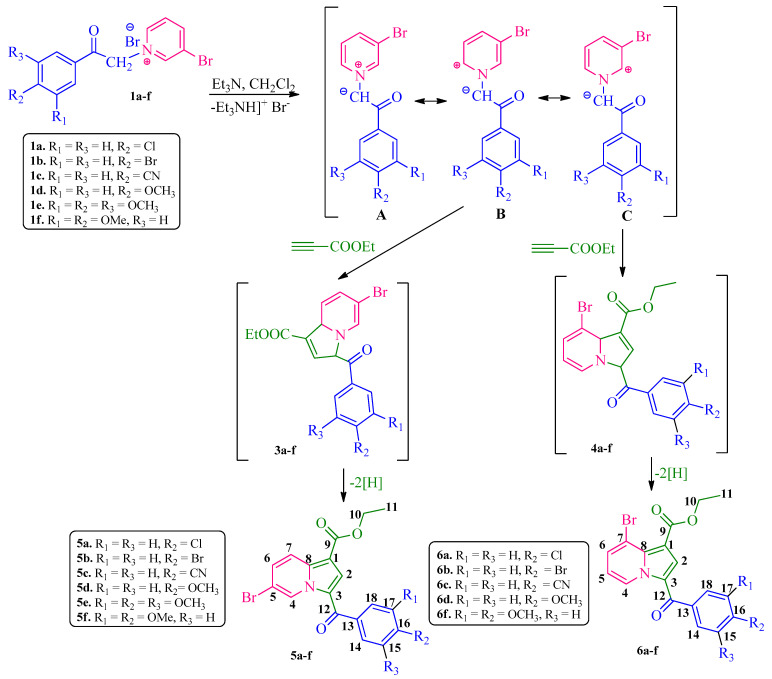
General pathway for the synthesis of cycloadducts **5a**–**f** and **6a**–**f** (**A**, **B** and **C** are resonance structures of the pyridinium ylides generated by salt type **1**).

The ylides generated by salts **1** can adopt two different resonance structures as 1,3-dipole (**B** and **C**) as can be seen in Scheme 2. Thus, the cycloaddition reactions of these ylides to ethyl propiolate (EP) can theoretically follow two different pathways. Although we anticipated the reaction to be regioselective due to the steric hindrance imposed by the bromine atom at the neighboring α-position in the ylide (in resonance structure **C**), we were surprised to observe the formation of both types of indolizine cycloadducts **5** and **6** in all reaction mixtures (Scheme 2). Probably the weak withdrawing effect of bromine played also a role in activating the ylide carbon and the hindrance is not strong enough to fully block the pathway to obtain compounds of type **6**. Derivatives **5** and **6** were isolated from the reaction mixture by crystallization and column chromatography (using dichloromethane as eluent) and subsequently purified by recrystallization using a dichloromethane/methanol mixture (1:2, *v*/*v*). Cycloadducts of type **5** were obtained in higher yields ranging from 37% to 30%, while compounds of type **6** were obtained in yields between 20% and 15%. In the case of salt **1e**, cycloadduct **5e**, even if observed on TLC, was impossible to isolate as a pure compound.

The lower yields obtained for the cycloaddition step reflect the formation of two types of cycloadducts. As the scope of our study was to investigate the potential of bromoindolizine, we prioritized compound access and characterization over reaction optimization. However, we anticipate that targeted adjustments (use of mild Lewis/π-acid activation, aromatization catalysts, different solvent, ultrasounds, etc.) can improve the yield and selectivity in subsequent studies.

From the reactions of ylides **B** and **C** with ethyl propiolate, only a single regioisomer has been isolated in accordance with the electronic effects of both the ylide and ethyl propiolate.

As mechanism, we presume that the 3+2 cycloaddition initially generates unstable intermediates **3** and **4**, which subsequently undergo oxidative aromatization to form the more stable aromatic derivatives **5** and **6** (Scheme 2).

The structures of the synthesized compounds were proven by elemental and spectral (IR, NMR, HRMS) methods and X-ray diffraction on monocrystal for compound **6a** (Supplementary Material contains ^1^H and ^13^C-NMR spectra of compounds **1c**, **5a**, **5c**, **6a**, **6c**, **7d**, **7g** (Figures S1–S14)).

The major structural differences between compounds of type **5** and **6** are in the ^1^H-NMR signals of pyridinic protons. Thus, in compounds of type **5**, protons H4 (Scheme 2) furnish a broad singlet or a doublet with a small coupling constant (*J* = 1.0 Hz), in the range of δ = 10.06–10.15 ppm, while in compounds of type **6**, the same proton 4 appears as a doublet (*J* = 7.0 Hz) or a doublet of doublets (dd) (*J* = 7.0; 0.5 Hz) in the range of δ = 9.90–10.02 ppm.

In compounds of type **5**, protons H6 appear as doublet (*J* = 9.5 Hz) or dd (*J* = 9.5; 1.5 Hz), sometimes overlapping with other signals, while the signal of protons H7 is a doublet (*J* = 9.0–9.5 Hz) located in the range of δ = 8.27–8.31 ppm.

In compounds of type **6**, protons H5 give rise to triplet signals (*J* = 7.5 Hz) at δ = 6.88–6.93 ppm, while the signals of protons H6 are observed as doublet (*J* = 7.0–7.5 Hz) or dd (*J* = 7.5; 1.5 Hz) at 7.62–7.71 ppm.

**Figure 2:**
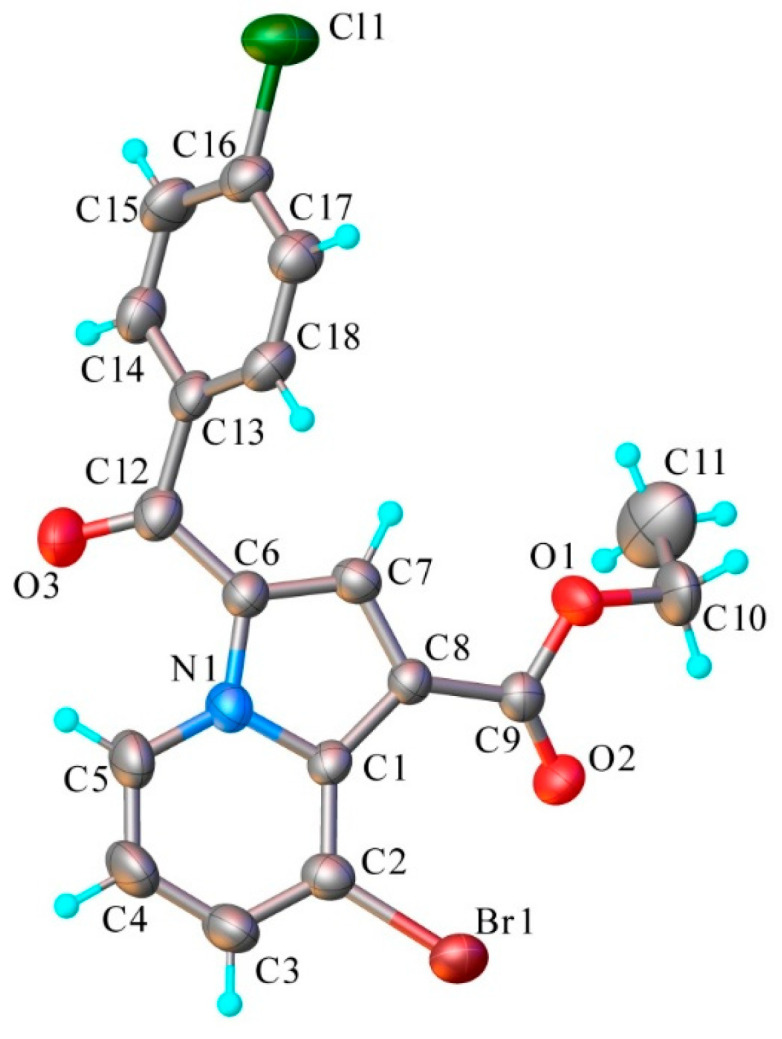
X-ray molecular structure of compound **6a** with atom labeling and thermal ellipsoids at 50% probability level.

The results of X-ray diffraction study for compound **6a** are illustrated in Figure 2, while refinement details and the geometric parameters are summarized in Tables S1 and S2 (Supplementary data). It exhibits a molecular crystal structure with one neutral unit in the asymmetric part of the unit cell. There are no co-crystallized interstitial molecules in the crystal. According to X-ray crystallography molecule **6a** is essentially non-planar, determining its helical structure. The indolizine fragment is twisted to avoid strong steric strain associated with short intramolecular contact C18-H···H-C7, so that the dihedral angle between indolizine and benzene rings reaches the value of 55.06°. This twist reduces overall planarity and highlights a conformational flexibility of the scaffold and positions the bromine substituent in a spatial orientation that could facilitate halogen bonding or hydrophobic contacts, which may influence intermolecular interactions and molecular recognition processes.

**Figure 3:**
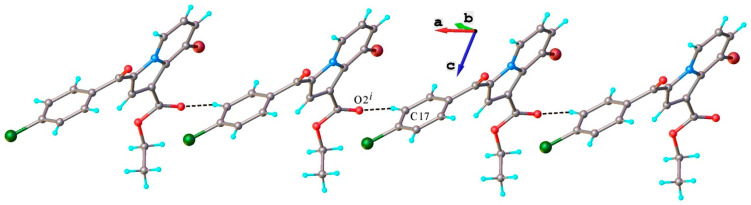
View of 1D supramolecular arrangement showing the role of C-H···O hydrogen binding. H bond parameters: C17-H···O2 [C17-H 0.93 Å, H···O2 (1 + x, y, z) 2.45 Å, C17···O2 3.368 (3) Å, ∠C17HO3 171.1°.

Analyzing the supramolecular features of the structure, taking into account the lack of conditions for classical hydrogen bonding, only weak C-H···O contacts were expected among the specific intermolecular interactions driving the crystal packing. Thus, the carbonyl O3 atom is involved in a short intermolecular C-H···O contact at 2.45 Å. In the crystal these contacts assemble the molecules into one-dimensional supramolecular arrays running along the *a* axis, as shown in Figure 3.

Although crystallization attempts with other derivatives were unsuccessful, the X-ray data for **6a** provide valuable conformational insights for the series. Full information concerning X-ray structure could be found in the Cambridge Crystallographic Data Centre (CCDC 2477072).

**Scheme 3:**
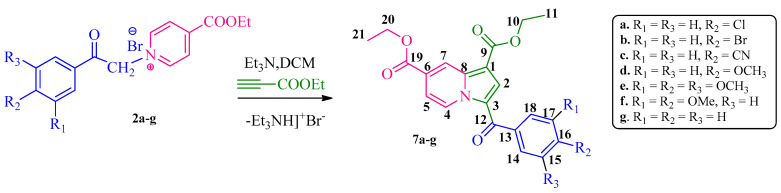
General pathway for the synthesis of cycloadducts **7a**–**g**.

When reaction conditions, comparable to those applied in the synthesis of compounds **5** and **6**, were used in case of salts **2a**–**g**, indolizines **7a**–**g** were obtained in moderate yields (Scheme 3). The spectral data also validate the proposed structures.

### 2.2. Biological Activity

#### 2.2.1. Anticancer Activity

All 31 synthesized compounds (13 monoquaternary salts and 18 cycloadducts) were submitted to the National Cancer Institute (NCI) platform for evaluation and 24 derivatives (12 salts (**1a**–**e**, **2a**–**g**) and 12 cycloadducts (**5a**–**d**, **6a**–**c**, **7a**–**d** and **7g**)) were tested for the anticancer properties at a single high dose (10^−5^ M) against a panel of 60 human tumor cell lines. These cell lines represent various cancer types, including leukemia, melanoma, and cancer of lung, colon, central nervous system, ovary, kidney, prostate and breast [28,29,30].

Representative results for the most active compounds are summarized in Table 1. All other tested compounds were inactive at 10^−5^ M in the NCI panel. Complete screening data for the active tested compounds (**1c**, **5a**, **5c**, **6a**, **6c**, **7d** and **7g**) are available in Supplementary Material Figures S15–S21.

Compounds **5c** (ethyl ester of 6-bromo-3-(4-cyanobenzoyl)indolizine-1-carboxylic acid) showed the strongest cytostatic activity across multiple cancer cell lines from various panels and also displayed cytotoxic effects against five cancer cell lines: HOP-62 and NCI-H226 (non-small cell lung cancer), SNB-75 (glioblastoma), SK-MEL-2 (melanoma), and RXF-393 (renal cancer).

Compounds **6c** (ethyl ester of 8-bromo-3-(4-cyanobenzoyl)indolizine-1-carboxylic acid) and **7g** (diethyl ester of 3-benzoylindolizine-1,7-dicarboxylic acid) also exhibited strong growth-inhibitory activity across multiple cancer cell lines, but with less cytotoxic effect (compound **6c** against SNB-75 glioblastoma cells and compound **7g** against HOP-62 lung cancer cells, respectively).

Compound **5a** showed moderate selective growth inhibition against non-small HOP-62 lung cancer cells, while compound **7d** had moderate effects in MOLT leukemia cells, NCI-H522 non-small cell lung cancer, and SK-MEL-5 melanoma cells.

Most of the monoquaternary salts were inactive, with the exception of compound **1c**, which displayed moderate activity against K-562 leukemia cells and HOP-62 lung cancer cells.

Interestingly, a majority of the active compounds demonstrated notable activity against the HOP-62 non-small cell lung cancer cell line, SNB-75 glioblastoma, suggesting a potential selectivity or enhanced sensitivity of this cell type to the tested indolizine derivatives.

#### 2.2.2. Molecular Docking

Molecular docking was performed to further investigate the binding interactions of the most promising indolizine compounds (**5c**, **6c**, **7g**). Considering the known ability of some indolizines to inhibit tubulin polymerization, and the fact that our group has previously reported fused pyrrolo-heterocyclic compounds with similar mechanisms of action [12,13,14,15,16,24], we selected tubulin as the molecular target for docking studies, focusing on the colchicine binding site.

The AutoDock results, followed by the analysis of 2D interaction diagrams, show how the ligands bind to the colchicine-binding site of tubulin, with some differences in the type of interactions and binding energy values. The 2D interaction diagrams provide a simplified view of how the ligands interact with the protein within a 4 Å cutoff, including important hydrogen or halogen bonds and electrostatic contacts. The interaction diagrams have a color code to indicate the nature of the interacting residues: red-orange for negatively charged residues, blue for positively charged, cyan for polar and green for hydrophobic. Purple arrows represent hydrogen bonds, while halogen bonds are represented with orange arrows. The direction of each “guitar-pick” symbol shows which part of the residue faces the ligand: if the pick points away from the ligand, the residue’s backbone is oriented toward it; if the pick points toward the ligand, the side chain is facing the ligand. Grey shading marks solvent-exposed regions. Table 2 summarizes the docked ligands, their lowest binding energy values, the corresponding binding conformations, and the associated 2D interaction diagrams. In the 3D representations, all ligands, including the reference compounds, are shown in CPK colors, hydrogen bonds are depicted as magenta dotted lines, while halogen bonds are also marked in orange. The tubulin residues interacting with the ligands are illustrated in stick representation.

Colchicine, the reference ligand, shows the most favorable binding energy (−10.08 kcal/mol), despite forming only one hydrogen bond with αVAL-181, as observed in our docking analysis. The binding with αVAL-181 is also visible in the X-ray structure of the tubulin–colchicine complex [31], suggesting that the docking results are consistent with experimental data. The relatively high affinity of colchicine may be attributed to hydrophobic anchoring and a high degree of shape complementarity with the protein pocket.

Phenstatin, also used as a reference ligand, forms a total of five hydrogen bonds—with αTHR-179, βGLN-247, βGLN-336, βLYS-352, and βTHR-353—but has a weaker binding energy (−8.04 kcal/mol) compared to colchicine. This extensive network of hydrogen bonds suggests a polar rather than hydrophobic anchoring, but not strong enough to match the affinity of colchicine.

Compound **5c** has the lowest binding energy among the synthetic ligands, −9.88 kcal/mol, outperforming phenstatin and only slightly weaker than colchicine. Its interaction profile is complex, including a hydrogen bond with the βASN-258 residue and two halogen bonds established between the bromine atom and the βALA-317 and βLYS-352 residues—an arrangement clearly illustrated in the 3D representation of Table 2. This set of interactions suggests that bromine contributes significantly to the stabilization of the complex through halogen bonding.

Compound **6c**, with a binding energy of −9.48 kcal/mol, ranks just after colchicine and **5c** in terms of binding affinity. Unlike **5c**, it does not form halogen bonds, but only a hydrogen bond with βASN-258, while maintaining a consistent hydrophobic anchorage in the protein pocket.

Compound **7g** has a different profile: it no longer forms hydrogen bonds with βASN-258, but establishes two alternative hydrogen bonds: one with αVAL-181 (residue also involved in the interaction with colchicine) and one with βLYS-254. This change in interaction partners suggests a possible repositioning of the ligand in the active site, while maintaining a network of contacts that gives it stability.

These findings are in agreement with the anticancer potency of compounds **5c**, **6c** and **7g**, suggesting that the cytotoxic effect may result from the interaction with colchicine-binding site of tubulin that usually lead to microtubule destabilization and mitotic arrest. This mode of action could explain the observed sensitivity in HOP-62 non-small cell lung cancer SNB-75 glioblastoma, both being characterized by relatively high proliferative rates and altered isotype expression, particularly βIII-tubulin [32,33]. While overexpressed βIII-tubulin has been associated with resistance to taxanes, colchicines-site ligands have been shown to retain activity in this context [32], which could explain the promising responses observed in case of our compounds.

#### 2.2.3. In Silico ADME [34] and Toxicity Predictions [35,36]

The pharmacokinetic, physicochemical and drug-likeness profile of the most active compounds **5c**, **6c** and **7g** was assessed using SwissADME web tool and the obtained data are presented in Table 3.

All three fulfilled Lipinski’s Rule of Five and Veber’s criteria, confirming good oral bioavailability potential.

Molecular weights (365.38–397.22 g/mol) are below the 500 g/mol threshold, while lipophilicity (Log Po/w = 2.26–2.37) indicates a good compromise between solubility and membrane permeability. The number of 4–5 hydrogen bond acceptors that also influences permeability is common in many approved small-molecules drugs. Compounds **5c**, **6c**, and **7g** do not contain H-bond donor groups, which comply with Lipinsky’s criterion of fewer than five. This absence can favor also membrane permeability, but at the same time, it can reduce water solubility and limit the number of hydrogen-bonding interactions with the target. Flexibility, reflected by 5–8 rotatable bonds, suggests that highly flexible molecule **7g** may engage diverse targets but at the expense of specificity, whereas rigid compounds like **5c** and **6c** may bind more selectively. Finally, TPSA values of 71.57–74.08 Å^2^ that lie well below 140 Å^2^ thresholds for oral bioavailability suggest good membrane permeability while maintaining sufficient polarity for solubility.

All three compounds are predicted to have high gastrointestinal (GI) absorption, indicating good potential for efficient uptake in the digestive tract. Also, predicted BBB (blood–brain barrier) permeability suggests possible CNS activity for these compounds. This property may increase the possibility of CNS-related off-target effects, which should be carefully considered in further pharmacological evaluations. At the same time, this is particularly relevant given the observed promising activity against glioblastoma SNB-75 cell line, since effective penetration of the BBB is necessary for CNS antitumor activity.

Additionally, none of the compounds are predicted to be P-glycoprotein (P-gp) substrates, which may contribute to enhanced bioavailability by reducing the likelihood of efflux-mediated drug resistance. Compounds **5c**, **6c** and **7g** exhibit moderate water solubility with a Log S value of in the range of −4.89–−5.44 and score well in terms of bioavailability (Abbott Bioavailability Score = 0.55) being within the range of developable drug candidates.

No PAINS or Brenk alerts in the structures of compounds **5c** and **6c**, while in case of compound **7g**, a Brenk alert flags the presence of two ester groups in its structure. These are typically flagged for their potential susceptibility to hydrolyze, causing a reduced metabolic stability. However, ester groups are frequently exploited in prodrug design to improve physicochemical properties such as solubility or membrane permeability. At the same time, many clinically approved drugs contain ester functionalities that remain stable under physiological conditions, demonstrating that such groups are not inherently unfavorable. Thus, while Brenk alert highlights a potential stability concern, it also provides an opportunity for a prodrug approach during lead optimization.

A synthetic accessibility score of 2.67–2.82 show the compounds present low synthetic complexity.

**Figure 4:**
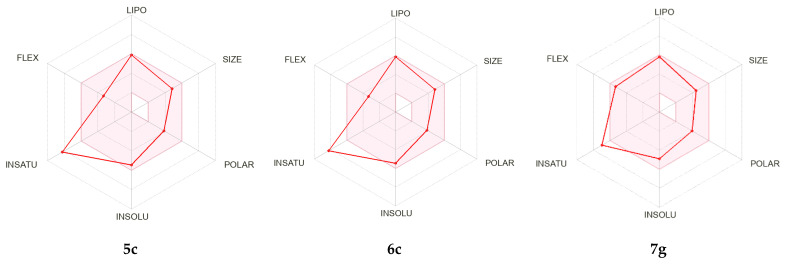
Bioavailability radar charts generated by SwissADME for compounds **5c**, **6c**, and **7g**.

The bioavailability radar (Figure 4) provides a visual overview of the drug-likeness of compounds **5c**, **6c** and **7g** by integrating six physicochemical parameters: lipophilicity (LIPO), size (SIZE), polarity (POLAR), solubility (INSOLU), unsaturation (INSATU), and flexibility (FLEX). As shown in Figure 4, compounds **5c**, **6c** and **7g** fit within the optimal (pink) region for five out of six parameters, with only one deviation observed for INSATU (ratio of sp^3^-hybridized carbons to total carbon atoms) parameter. This deviation indicates a somewhat higher degree of planarity compared to the average of orally active drugs, but it does not represent a critical limitation, as many approved drugs display similar trends. Overall, the radar highlights that our compounds maintain a favorable balance of properties predictive of good oral bioavailability, thereby supporting their potential as drug-like candidates.

Toxicity predictions for the three lead compounds are summarized in Table 4. The predicted toxicity profile of compounds **5c**, **6c** and **7g** expressed as a list of activities with associated probabilities of being active (Pa) or inactive (Pi) was conducted against 391 tumor cell lines and 47 normal human cell lines representing diverse tissue origins. As shown in Table 4, the results indicate predicted cytotoxicity (Pa > Pi and Pa > 0.5 that represent a moderate probability to be active) against hepatocellular carcinoma SNU-398 and glioblastoma SNB-75. The predicted activity of compounds **5c**, **6c** and **7g** against glioblastoma SNB-75 is consistent with the NCI screening results which demonstrated cytotoxic effect at 10^−5^ M against the same cell line (total growth inhibition with cytotoxic effect for compounds **5c** and **6c** and 64% inhibition in case of compound **7g**). The absence of normal human cell lines from the prediction list may suggest a favorable selectivity of the tested compounds toward cancer cells.

## 3. Materials and Methods

### 3.1. Chemistry

All commercially available reagents and solvents were used without further purification. Thin-layer chromatography (TLC) was performed on Merck 60F_254_ silica gel plates (Merck, Darmstadt, Germany). Visualization of the plates was achieved using a UV lamp (λ_max_ = 254 nm or 365 nm). Melting points were recorded on an A. Krüss Optronic Melting Point Meter KSP1N (Kruss, Hamburg, Germany) and are uncorrected. Proton and carbon nuclear magnetic resonance (NMR) spectra were recorded on a Bruker Avance III 500 MHz spectrometer (500 MHz, Bruker BioSpin GmbH, Rheinstetten, Germany), using CDCl_3_ or DMSO as internal standards. Chemical shifts (δ) are reported in part per million (ppm) and coupling constants (*J*) in Hz. The following abbreviations are used to designate chemical shift multiplicities: s = singlet, d = doublet, t = triplet, q = quartet, m = multiplet, bs = broad singlet, dd = doublet of doublets. Infrared (IR) spectra were recorded as films with preparation performed by pelletizing with potassium bromide (KBr), on a Jasco 660 *plus* FTIR spectrophotometer. Elemental analyses indicated by the symbols of the elements were within ±0.4% of the theoretical values. HRMS experiments were recorded on an ESI -Orbitrap Exploris 120 Mass Spectrometer (Thermo Fisher, Waltham, MA, USA) in positive mode.

#### 3.1.1. General Procedure for Quaternary Salts **1** and **2**

A solution of 3-bromopyridine/ethyl isonicotinate (5 mmol, 1 eq.) and the corresponding 2-bromoacetophenone (5.5 mmol, 1.1 eq.) was prepared by dissolving the reagents in a minimal volume of acetone (7–10 mL) and subjected to magnetic stirring for 6 h at room temperature. Then, the formed salt that appears as a cream precipitate was separated by filtration and washed with acetone. After drying, the compound was used in the subsequent steps without requiring further purification.

#### 3.1.2. General Procedure for Compounds **5**, **6**, **7**

The cycloimmonium salt (**1** or **2**) (1 mmol, 1 eq.) was suspended in 10 mL of dichloromethane, followed by the addition of ethyl propiolate (1.1 mmol, 1.1 eq.). Triethylamine (Et_3_N) (3 eq.) was added dropwise over approximately 20 min. During that time, the solution’s color turned orange/yellow, indicating the formation of the corresponding ylide. The obtained reaction mixture was magnetic stirred overnight at room temperature. Then, approximately 7 mL of methanol was added, and the mixture was left in a crystallization dish overnight without stirring. The resulting precipitate was filtered and the obtained solid was identified as compound **5** (in case of salts type **1**) or **7** (in case of salts type **2**). The remaining solutions were subjected to column chromatography using dichloromethane as eluent to obtain compounds **5** (very small quantities) and **6** (in case of salts type **1**) or compound **7** (only small quantities, in case of salts type **2**). The purification of the separated compounds was achieved by recrystallization from dichloromethane/methanol mixture (1:2, *v*/*v*).

#### 3.1.3. Spectral Data

3-bromo-1-(2-(4-chlorophenyl)-2-oxoethyl)pyridin-1-ium bromide **1a**. White solid, yield = 87%, m.p. = 231–232 °C. IR (KBr), ν (cm^−1^): 3069, 2989, 2922, 1696, 1589, 1483, 1238, 1181, 997, 819. ^1^H NMR (DMSO-d_6_, 500 MHz), δ (ppm): 6.50 (s, 2H, H7), 7.76 (d, *J* = 8.5 Hz, 2H, H11, H13), 8.08 (d, *J* = 8.5 Hz, 2H, H10, H14), 8.27 (dd, *J* = 8.5; 6.0 Hz, 1H, H5), 9.04 (d, *J* = 8.5 Hz, 1H, H4), 9.07 (d, *J* = 6.0 Hz, 1H, H6), 9.46 (s, 1H, H2). ^13^C NMR (DMSO-d_6_, 125 MHz), δ (ppm): 66.2 (C7), 121.5 (C3), 128.7 (C5), 129.4 (C11, C13), 130.2 (C10, C14), 132.2 (C9), 139.7 (C12), 145.5 (C6), 147.4 (C2), 148.9 (C4), 189.5 (C8). Anal. Calcd. for C_13_H_10_Br_2_ClNO: C, 39.88; H, 2.57; N, 3.58. Found: C, 39.87; H, 2.55; N, 3.59.

3-bromo-1-(2-(4-bromophenyl)-2-oxoethyl)pyridin-1-ium bromide **1b**. White solid, yield = 77%, m.p. = 239–240 °C. IR (KBr), ν (cm^−1^): 3068, 2988, 2922, 1695, 1584, 1483, 1237, 1180, 1071, 994, 817. ^1^H NMR (DMSO-d_6_, 500 MHz), δ (ppm): 6.47 (s, 2H, H7), 7.90 (d, *J* = 8.5 Hz, 2H, H11, H13), 8.99 (d, *J* = 8.5 Hz, 2H, H10, H14), 8.26 (dd, J = 8.5; 6.0 Hz, 1H, H5), 9.04 (d, *J* = 9.0 Hz, 1H, H4), 9.06 (d, *J* = 6.0 Hz, 1H, H6), 9.44 (s, 1H, H2). ^13^C NMR (DMSO-d_6_, 125 MHz), δ (ppm): 66.2 (C7), 121.5 (C3), 128.7 (C5), 129.0 (C12), 130.2 (C10, C14), 132.3 (C11, C13), 132.5 (C9), 145.5 (C6), 147.4 (C2), 148.9 (C4), 189.7 (C8). Anal. Calcd. for C_13_H_10_Br_3_NO: C, 35.82; H, 2.31; N, 3.21. Found: C, 35.83; H, 2.30; N, 3.23.

3-bromo-1-(2-(4-cyanophenyl)-2-oxoethyl)pyridin-1-ium bromide **1c**. White solid, yield = 75%, m.p. = 234–235 °C. IR (KBr), ν (cm^−1^): 3025, 2993, 2920, 2231, 1706, 1492, 1458, 1352, 1234, 1215, 997. ^1^H NMR (DMSO-d_6_, 500 MHz), δ (ppm): 6.54 (s, 2H, H7), 7.17 (d, *J* = 8.5 Hz, 2H, H11, H13), 8.22 (d, *J* = 8.5 Hz, 2H, H10, H14), 8.27 (dd, *J* = 8.5; 6.0 Hz, 1H, H5), 9.05 (d, *J* = 9.0 Hz, 1H, H4), 9.07 (d, *J* = 6.5 Hz, 1H, H6), 9.46 (s, 1H, H2). ^13^C NMR (DMSO-d_6_, 125 MHz), δ (ppm): 66.4 (C7), 116.4 (C12), 118.0 (C15), 121.5 (C3), 128.8 (C5), 128.9 (C10, C14), 133.2 (C11, C13), 136.7 (C9), 145.5 (C6), 147.3 (C2), 149.0 (C4), 189.9 (C8). Anal. Calcd. for C_14_H_10_Br_2_N_2_O: C, 44.01; H, 2.64; N, 7.33. Found: C, 44.00; H, 2.62; N, 7.34.

3-bromo-1-(2-(4-methoxyphenyl)-2-oxoethyl)pyridin-1-ium bromide **1d**. White solid, yield = 85%, m.p. = 230–231 °C. IR (KBr), ν (cm^−1^): 3064, 2980, 2925, 1677, 1604, 1482, 1425, 1349, 1249, 1177, 1030. ^1^H NMR (DMSO-d_6_, 500 MHz), δ (ppm): 3.90 (s, 3H, H15), 6.43 (s, 2H, H7), 7.19 (d, *J* = 8.5 Hz, 2H, H11, H13), 8.04 (d, *J* = 8.5 Hz, 2H, H10, H14), 8.25 (dd, *J* = 8.0; 6.0 Hz, 1H, H5), 9.02 (d, *J* = 8.5 Hz, 1H, H4), 9.07 (d, *J* = 6.0 Hz, 1H, H6), 9.46 (s, 1H, H2). ^13^C NMR (DMSO-d_6_, 125 MHz), δ (ppm): 55.9 (C15), 66.0 (C7), 114.5 (C11, C13), 121.5 (C3), 126.2 (C9), 128.6 (C5), 130.8 (C10, C14), 145.5 (C6), 147.4 (C2), 148.7 (C4), 164.4 (C12), 188.5 (C8). Anal. Calcd. for C_14_H_13_Br_2_NO_2_: C, 43.44; H, 3.39; N, 3.62. Found: C, 43.45; H, 3.37; N, 3.64.

3-bromo-1-(2-(3,4,5-trimethoxyphenyl)-2-oxoethyl)pyridin-1-ium bromide **1e**. White solid, yield = 60%, m.p. = 194–195 °C. IR (KBr), ν (cm^−1^): 3076, 2939, 2913, 1679, 1584, 1461, 1417, 1347, 1165, 1128, 991. ^1^H NMR (DMSO-d_6_, 500 MHz), δ (ppm): 3.80 (s, 3H, OMe), 3.90 (s, 6H, 2 × OMe), 6.51 (s, 2H, H7), 7.36 (s, 2H, H10, H14), 8.26 (dd, *J* = 8.0; 6.0 Hz, 1H, H5), 9.03 (d, *J* = 8.5 Hz, 1H, H4), 9.07 (d, *J* = 6.0 Hz, 1H, H6), 9.46 (s, 1H, H2). ^13^C NMR (DMSO-d_6_, 125 MHz), δ (ppm): 56.4 (C15, C17), 60.4 (C16), 66.3 (C7), 106.1 (C10, C14), 121.6 (C3), 128.6 (C5), 128.8 (C9), 143.2 (C12), 145.4 (C6), 147.4 (C2), 148.9 (C4), 153.1 (C11, C13), 189.2 (C8). Anal. Calcd. for C_16_H_17_Br_2_NO_4_: C, 42.98; H, 3.83; N, 3.13. Found: C, 42.98; H, 3.81; N, 3.14.

3-bromo-1-(2-(3,4-dimethoxyphenyl)-2-oxoethyl)pyridin-1-ium bromide **1f**. Cream solid, yield = 50%, m.p. = 209–210 °C. IR (KBr), ν (cm^−1^): 3060, 2996, 1683, 1584, 1518, 1419, 1267, 1149, 1011. ^1^H NMR (DMSO-d_6_, 500 MHz), δ (ppm): 3.85 (s, 3H, H15), 3.91 (s, 3H, H16), 6.47 (s, 2H, H7), 7.23 (d, *J* = 8.5 Hz, 1H, H13), 7.51 (d, *J* = 2.0 Hz, 1H, H10), 7.76 (dd, *J* = 8.5; 2.0 Hz, 1H, H14), 8.25 (dd, *J* = 8.5; 6.0 Hz, 1H, H5), 9.02 (d, *J* = 8.5 Hz, 1H, H4), 9.06 (d, *J* = 6.0 Hz, 1H, H6), 9.45 (s, 1H, H2). ^13^C NMR (DMSO-d_6_, 125 MHz), δ (ppm): 55.8 (C15), 56.0 (C16), 66.0 (C7), 110.2 (C10), 111.3 (C13), 121.5 (C3), 123.5 (C14), 126.1 (C9), 128.7 (C5), 145.5 (C6), 147.4 (C2), 148.8 (C11), 148.9 (C4), 154.4 (C12), 188.5 (C8). Anal. Calcd. for C_15_H_15_Br_2_NO_3_: C, 43.19; H, 3.62; N, 3.36. Found: C, 43.17; H, 3.61; N, 3.37.

1-(2-(4-chlorophenyl)-2-oxoethyl)-4-(ethoxycarbonyl)pyridin-1-ium bromide **2a**. White solid, yield = 83%, m.p. = 171–172 °C. IR (KBr), ν (cm^−1^): 3019, 2987, 2935, 1732, 1694, 1583, 1475, 1299, 1220, 1142, 1089, 992. ^1^H NMR (DMSO-d_6_, 500 MHz), δ (ppm): 1.39 (t, *J* = 7.0 Hz, 3H, H17), 4.46 (q, *J* = 7.0 Hz, 2H, H16), 6.63 (s, 2H, H7), 7.76 (d, *J* = 8.5 Hz, 2H, H11, H13), 8.09 (d, *J* = 8.5 Hz, 2H, H10, H14), 8.66 (d, *J* = 7.0 Hz, 2H, H3, H5), 9.22 (d, *J* = 7.0 Hz, 2H, H2, H6). ^13^C NMR (DMSO-d_6_, 125 MHz), δ (ppm): 13.9 (C17), 63.0 (C16), 66.6 (C7), 127.0 (C3, C5), 129.3 (C11, C13), 130.2 (C10, C14), 132.3 (C9), 139.6 (C12), 144.8 (C4), 147.8 (C2, C6), 162.0 (C15), 189.6 (C8). HRMS (ESI, positive mode): *m*/*z* calcd for C_16_H_15_ClNO_3_^+^ [M-Br]^+^: 304.0735; found: 304.0735. Anal. Calcd. for C_16_H_15_BrClNO_3_: C, 49.96; H, 3.93; N, 3.64. Found: C, 49.96; H, 3.92; N, 3.66.

1-(2-(4-bromophenyl)-2-oxoethyl)-4-(ethoxycarbonyl)pyridin-1-ium bromide **2b**. White solid, yield = 60%, m.p. = 203–204 °C. IR (KBr), ν (cm^−1^): 3013, 2993, 2973, 1734, 1692, 1585, 1468, 1296, 1237, 1137, 988. ^1^H NMR (DMSO-d_6_, 500 MHz), δ (ppm): 1.39 (t, *J* = 7.0 Hz, 3H, H17), 4.47 (q, *J* = 7.0 Hz, 2H, H16), 6.63 (s, 2H, H7), 7.90 (d, *J* = 8.5 Hz, 2H, H11, H13), 8.00 (d, *J* = 8.5 Hz, 2H, H10, H14), 8.66 (d, *J* = 7.0 Hz, 2H, H3, H5), 9.22 (d, *J* = 7.0 Hz, 2H, H2, H6). ^13^C NMR (DMSO-d_6_, 125 MHz), δ (ppm): 13.9 (C17), 63.0 (C16), 66.6 (C7), 127.0 (C3, C5), 128.9 (C12), 130.3 (C10, C14), 132.3 (C11, C13), 132.6 (C9), 144.8 (C4), 147.8 (C2, C6), 162.0 (C15), 189.8 (C8). Anal. Calcd. for C_16_H_15_Br_2_NO_3_: C, 44.78; H, 3.52; N, 3.26. Found: C, 44.76; H, 3.51; N, 3.27.

1-(2-(4-cyanophenyl)-2-oxoethyl)-4-(ethoxycarbonyl)pyridin-1-ium bromide **2c**. White solid, yield = 98%, m.p. = 202–204 °C. IR (KBr), ν (cm^−1^): 3021, 2990, 2936, 2228, 1732, 1699, 1578, 1474, 1303, 1219, 1142, 995. ^1^H NMR (DMSO-d_6_, 500 MHz), δ (ppm): 1.39 (t, *J* = 7.0 Hz, 3H, H17), 4.47 (q, *J* = 7.0 Hz, 2H, H16), 6.68 (s, 2H, H7), 7.17 (d, *J* = 8.5 Hz, 2H, H11, H13), 8.22 (d, *J* = 8.0 Hz, 2H, H10, H14), 8.68 (d, *J* = 7.0 Hz, 2H, H3, H5), 9.23 (d, *J* = 7.0 Hz, 2H, H2, H6). ^13^C NMR (DMSO-d_6_, 125 MHz), δ (ppm): 13.9 (C17), 63.0 (C16), 66.9 (C7), 116.3 (C12), 118.0 (C18), 127.1 (C3, C5), 128.9 (C10, C14), 133.2 (C11, C13), 136.8 (C9), 144.8 (C4), 147.8 (C2, C6), 162.0 (C15), 190.0 (C8). Anal. Calcd. for C_17_H_15_BrN_2_O_3_: C, 54.42; H, 4.03; N, 7.47. Found: C, 54.41; H, 4.02; N, 7.49.

1-(2-(4-methoxyphenyl)-2-oxoethyl)-4-(ethoxycarbonyl)pyridin-1-ium bromide **2d**. White solid, yield = 85%, m.p. = 198–199 °C. IR (KBr), ν (cm^−1^): 3019, 2986, 2935, 1735, 1680, 1599, 1575, 1299, 1268, 1233, 1182, 1023, 849. ^1^H NMR (DMSO-d_6_, 500 MHz), δ (ppm): 1.39 (t, *J* = 7.0 Hz, 3H, H17), 3.90 (s, 3H, H18), 4.46 (q, *J* = 7.0 Hz, 2H, H16), 6.60 (s, 2H, H7), 7.19 (d, *J* = 8.5 Hz, 2H, H11, H13), 8.05 (d, *J* = 8.5 Hz, 2H, H10, H14), 8.65 (d, *J* = 6.0 Hz, 2H, H3, H5), 9.23 (d, *J* = 6.5 Hz, 2H, H2, H6). ^13^C NMR (DMSO-d_6_, 125 MHz), δ (ppm): 13.9 (C17), 55.9 (C18), 63.0 (C16), 66.4 (C7), 114.5 (C11, C13), 126.3 (C9), 126.9 (C3, C5), 130.8 (C10, C14), 144.6 (C4), 147.8 (C2, C6), 162.1 (C15), 164.3 (C12), 188.6 (C8). Anal. Calcd. for C_17_H_18_BrNO_4_: C, 53.70; H, 4.77; N, 3.68. Found: C, 53.68; H, 4.76; N, 3.70.

1-(2-(3,4,5-trimethoxyphenyl)-2-oxoethyl)-4-(ethoxycarbonyl)pyridin-1-ium bromide **2e**. Yellow solid, yield = 70%, m.p. = 200–201 °C. IR (KBr), ν (cm^−1^): 3033, 2978, 2936, 1729, 1682, 1581, 1464, 1334, 1288, 1165, 1119, 1001. ^1^H NMR (DMSO-d_6_, 500 MHz), δ (ppm): 1.39 (t, *J* = 7.0 Hz, 3H, H17), 3.80 (s, 3H, OMe), 3.90 (s, 6H, 2 × OMe), 4.47 (q, *J* = 7.0 Hz, 2H, H16), 6.67 (s, 2H, H7), 7.37 (s, 2H, H10, H14), 8.66 (d, *J* = 6.5 Hz, 2H, H3, H5), 9.22 (d, *J* = 6.5 Hz, 2H, H2, H6). ^13^C NMR (DMSO-d_6_, 125 MHz), δ (ppm): 13.9 (C17), 56.4 (2 × OMe), 60.4 (OMe), 63.0 (C16), 66.7 (C7), 106.1 (C10, C14), 127.1 (C3, C5), 128.7 (C9), 143.2 (C12), 144.8 (C4), 147.7 (C2, C6), 153.0 (C11, C13), 162.0 (C15), 189.3 (C8). Anal. Calcd. for C_19_H_22_BrNO_6_: C, 51.83; H, 5.04; N, 3.18. Found: C, 51.84; H, 5.02; N, 3.19.

1-(2-(3,4-dimethoxyphenyl)-2-oxoethyl)-4-(ethoxycarbonyl)pyridin-1-ium bromide **2f**. White solid, yield = 83%, m.p. = 221–223 °C. IR (KBr), ν (cm^−1^): 3087, 3028, 2942, 2891, 1727, 1678, 1595, 1582, 1463, 1347, 1277, 1211, 1139, 1017, 752. ^1^H NMR (DMSO-d_6_, 500 MHz), δ (ppm): 1.39 (t, *J* = 7.0 Hz, 3H, H17), 3.85 (s, 3H, OMe), 3.91 (s, 3H, OMe), 4.47 (q, *J* = 7.0 Hz, 2H, H16), 6.59 (s, 2H, H7), 7.23 (d, *J* = 8.5 Hz, 1H, H13), 7.51 (d, *J* = 2.0 Hz, 1H, H10), 7.76 (dd, *J* = 8.5; 2.0 Hz, 1H, H14), 8.65 (d, *J* = 6.5 Hz, 2H, H3, H5), 9.21 (d, *J* = 6.5 Hz, 2H, H2, H6). ^13^C NMR (DMSO-d_6_, 125 MHz), δ (ppm): 13.9 (C17), 55.8 (OMe), 56.0 (OMe), 63.0 (C16), 66.4 (C7), 110.3 (C10), 111.3 (C13), 123.5 (C14), 126.2 (C9), 127.0 (C3, C5), 144.7 (C4), 147.8 (C2, C6), 148.9 (C11), 154.4 (C12), 162.0 (C15), 188.6 (C8). Anal. Calcd. for C_18_H_20_BrNO_5_: C, 52.70; H, 4.91; N, 3.41. Found: C, 52.69; H, 4.90; N, 3.43.

4-(ethoxycarbonyl)-1-(2-oxo-2-phenylethyl)pyridin-1-ium bromide **2g**. White solid, yield = 60%, m.p. = 184–185 °C. IR (KBr), ν (cm^−1^): 3099, 3029, 2938, 1728, 1690, 1451, 1275, 1116, 757. ^1^H NMR (DMSO-d_6_, 500 MHz), δ (ppm): 1.39 (t, *J* = 7.0 Hz, 3H, H17), 4.47 (q, *J* = 7.0 Hz, 2H, H16), 6.65 (s, 2H, H7), 7.67 (t, *J* = 8.0 Hz, 2H, H11, H13), 7.80 (t, *J* = 7.5 Hz, 1H, H12), 8.08 (d, *J* = 7.5 Hz, 2H, H10, H14), 8.66 (d, *J* = 7.0 Hz, 2H, H3, H5), 9.24 (d, *J* = 7.0 Hz, 2H, H2, H6). ^13^C NMR (DMSO-d_6_, 125 MHz), δ (ppm): 13.9 (C17), 63.0 (C16), 66.7 (C7), 127.0 (C3, C5), 128.3 (C10, C14), 129.2 (C11, C13), 133.5 (C9), 134.8 (C12), 144.7 (C4), 147.8 (C2, C6), 162.0 (C15), 190.4 (C8). Anal. Calcd. for C_16_H_16_BrNO_3_: C, 54.87; H, 4.61; N, 4.00. Found: C, 54.87; H, 4.60; N, 4.02.

Ethyl ester of 6-bromo-3-(4-chlorobenzoyl)indolizine-1-carboxylic acid **5a**. Yellow solid, yield = 30%, m.p. = 138–139 °C. IR (KBr), ν (cm^−1^): 3118, 2973, 1704, 1620, 1515, 1475, 1356, 1215, 1056, 751. ^1^H NMR (CDCl_3_, 500 MHz), δ (ppm): 1.40 (t, *J* = 7.0 Hz, 3H, H11), 4.37 (q, *J* = 7.0 Hz, 2H, H10), 7.49–7.52 (overlapping signals, 3H, H15, H17, H6), 7.74–7.77 (overlapping signals, 3H, H2, H14, H18), 8.29 (d, *J* = 9.0 Hz, 1H, H7), 10.11 (s, 1H, H4). ^13^C NMR (CDCl_3_, 125 MHz), δ (ppm): 14.7 (C11), 60.6 (C10), 107.4 (C1), 110.7 (C5), 120.2 (C7), 122.3 (C3), 128.5 (C2), 129.0 (C15, C17), 129.3 (C4), 131.1 (C6), 130.5 (C14, C18), 137.9 (C13), 138.1 (C8), 138.3 (C16), 163.7 (C9), 184.3 (C12). Anal. Calcd. for C_18_H_13_BrClNO_3_: C, 53.16; H, 3.22; N, 3.44. Found: C, 53.15; H, 3.20; N, 3.45.

Ethyl ester of 6-bromo-3-(4-bromobenzoyl)indolizine-1-carboxylic acid **5b**. Pale yellow solid, yield = 33%, m.p. = 160–161 °C. IR (KBr), ν (cm^−1^): 3093, 2966, 2927, 1706, 1623, 1517, 1476, 1356, 1213, 1054, 811. ^1^H NMR (CDCl_3_, 500 MHz), δ (ppm): 1.40 (t, *J* = 7.0 Hz, 3H, H11), 4.37 (q, *J* = 7.0 Hz, 2H, H10), 7.52 (dd, *J* = 9.5; 1.5 Hz, 1H, H6), 7.66 (d, *J* = 9.0 Hz, 2H, H15, H17), 7.69 (d, *J* = 9.0 Hz, 2H, H14, H18), 7.74 (s, 1H, H2), 8.29 (d, *J* = 9.0 Hz, 1H, H7), 10.11 (d, *J* = 1.0 Hz, 1H, H4). ^13^C NMR (CDCl_3_, 125 MHz), δ (ppm): 14.7 (C11), 60.6 (C10), 107.4 (C1), 110.8 (C5), 120.2 (C7), 122.3 (C3), 126.8 (C16), 128.5 (C2), 129.3 (C4), 130.6 (C14, C18), 131.1 (C6), 131.9 (C15, C17), 138.2 (C8), 138.3 (C13), 163.7 (C9), 184.4 (C12). HRMS (ESI, positive mode): *m*/*z* calcd for C_18_H_13_Br_2_NO_3_ [M^+^]: 448.9262; found: 448.9259. Anal. Calcd. for C_18_H_13_Br_2_NO_3_: C, 47.92; H, 2.90; N, 3.10. Found: C, 47.91; H, 2.88; N, 3.12.

Ethyl ester of 6-bromo-3-(4-cyanobenzoyl)indolizine-1-carboxylic acid **5c**. Yellow solid, yield = 35%, m.p. = 202–203 °C. IR (KBr), ν (cm^−1^): 3096, 2976, 2927, 2224, 1705, 1616, 1521, 1473, 1355, 1259, 1219, 1041, 817. ^1^H NMR (CDCl_3_, 500 MHz), δ (ppm): 1.40 (t, *J* = 7.0 Hz, 3H, H11), 4.38 (q, *J* = 7.0 Hz, 2H, H10), 7.57 (dd, *J* = 9.5; 1.5 Hz, 1H, H6), 7.70 (s, 1H, H2), 7.83 (d, *J* = 8.5 Hz, 2H, H15, H17), 7.88 (d, *J* = 8.5 Hz, 2H, H14, H18), 8.31 (d, *J* = 9.0 Hz, 1H, H7), 10.15 (s, 1H, H4). ^13^C NMR (CDCl_3_, 125 MHz), δ (ppm): 14.6 (C11), 60.7 (C10), 107.9 (C1), 111.2 (C5), 115.3 (C16), 118.2 (CN), 120.3 (C7), 122.0 (C3), 128.8 (C2), 129.4 (C4), 129.5 (C14, C18), 131.7 (C6), 132.5 (C15, C17), 138.5 (C8), 143.3 (C13), 163.5 (C9), 183.5 (C12). Anal. Calcd. for C_19_H_13_BrN_2_O_3_: C, 57.45; H, 3.30; N, 7.05. Found: C, 57.44; H, 3.29; N, 7.07.

Ethyl ester of 6-bromo-3-(4-methoxybenzoyl)indolizine-1-carboxylic acid **5d**. Brown solid, yield = 37%, m.p. = 149–150 °C. IR (KBr), ν (cm^−1^): 3094, 2977, 2938, 1699, 1613, 1599, 1518, 1477, 1356, 1265, 1222, 1173, 1059, 1025. ^1^H NMR (CDCl_3_, 500 MHz), δ (ppm): 1.40 (t, *J* = 7.0 Hz, 3H, H11), 3.91 (s, 3H, H19), 4.38 (q, *J* = 7.0 Hz, 2H, H10), 7.02 (d, *J* = 8.5 Hz, 2H, H15, H17), 7.47 (dd, *J* = 9.5; 1.5 Hz, 1H, H6), 7.79 (s, 1H, H2), 7.84 (d, *J* = 8.5 Hz, 2H, H14, H18), 8.27 (d, *J* = 9.0 Hz, 1H, H7), 10.08 (d, *J* = 1.0 Hz, 1H, H4). ^13^C NMR (CDCl_3_, 125 MHz), δ (ppm): 14.7 (C11), 55.7 (C19), 60.4 (C10), 106.8 (C1), 110.3 (C5), 113.9 (C15, C17), 120.1 (C7), 122.8 (C3), 128.0 (C2), 129.2 (C4), 130.5 (C6), 131.4 (C14, C18), 132.0 (C13), 137.8 (C8), 162.9 (C16), 164.0 (C9), 184.7 (C12). Anal. Calcd. for C_19_H_16_BrNO_4_: C, 56.73; H, 4.01; N, 3.48. Found: C, 56.71; H, 4.00; N, 3.49.

Ethyl ester of 6-bromo-3-(3,4,5-trimethoxybenzoyl)indolizine-1-carboxylic acid **5e**. White solid, yield = 30%, m.p. = 248–249 °C. IR (KBr), ν (cm^−1^): 3074, 2983, 1702, 1619, 1583, 1520, 1476, 1360, 1339, 1221, 1135, 1050, 753. ^1^H NMR (CDCl_3_, 500 MHz), δ (ppm): 1.40 (t, *J* = 7.0 Hz, 3H, H11), 3.92 (s, 6H, 2 × OCH_3_, H21), 3.96 (s, 3H, OCH_3_), 4.38 (q, *J* = 7.0 Hz, 2H, H10), 7.07 (s, 2H, H14, H18), 7.51 (d, *J* = 9.5 Hz, 1H, H6), 7.85 (s, 1H, H2), 8.30 (d, *J* = 9.5 Hz, 1H, H7), 10.09 (s, 1H, H4). ^13^C NMR (CDCl_3_, 125 MHz), δ (ppm): 14.7 (C11), 56.5 (C19, C21), 60.4 (C10), 61.1 (C20), 106.8 (C14, C18), 107.2 (C1), 110.6 (C5), 120.2 (C7), 122.5 (C3), 128.4 (C2), 129.3 (C4), 130.8 (C6), 134.7 (C13), 138.0 (C8), 153.22 (C15, C17), 141.6 (C16), 163.9 (C9), 184.8 (C12). Anal. Calcd. for C_21_H_20_BrNO_6_: C, 54.56; H, 4.36; N, 3.03. Found: C, 54.55; H, 4.35; N, 3.04.

Ethyl ester of 6-bromo-3-(3,4-dimethoxybenzoyl)indolizine-1-carboxylic acid **5f**. Brown solid, yield = 32%, m.p. = 123–124 °C. IR (KBr), ν (cm^−1^): 3078, 3016, 2899, 1702, 1627, 1516, 1479 1266, 1201, 1174, 1029, 747. ^1^H NMR (CDCl_3_, 500 MHz), δ (ppm): 1.40 (t, *J* = 7.0 Hz, 3H, H11), 3.97 (s, 3H, OCH_3_), 3.99 (s, 3H, OCH_3_), 4.38 (q, *J* = 7.0 Hz, 2H, H10), 6.98 (d, *J* = 8.5 Hz, 1H, H17), 7.42 (d, *J* = 2.0 Hz, 1H, H14), 7.47–7.49 (overlapping signals, 2H, H18, H6), 7.82 (s, 1H, H2), 8.28 (d, *J* = 9.5 Hz, 1H, H7), 10.06 (s, 1H, H4). ^13^C NMR (CDCl_3_, 125 MHz), δ (ppm): 14.7 (C11), 56.2 (C19), 56.3 (C20), 60.4 (C10), 106.9 (C1), 110.2 (C17), 110.3 (C5), 111.7 (C14), 120.1 (C7), 122.8 (C3), 123.7 (C18), 128.0 (C2), 129.2 (C4), 130.5 (C6), 132.1 (C13), 137.8 (C8), 149.3 (C15), 152.6 (C16), 164.0 (C9), 184.6 (C12). Anal. Calcd. for C_20_H_18_BrNO_5_: C, 55.57; H, 4.20; N, 3.24. Found: C, 55.56; H, 4.19; N, 3.25.

Ethyl ester of 8-bromo-3-(4-chlorobenzoyl)indolizine-1-carboxylic acid **6a**. Yellow crystals, yield = 15%, m.p. = 160–161 °C. IR (KBr), ν (cm^−1^): 3099, 2983, 1737, 1623, 1525, 1473, 1357, 1165, 1089. ^1^H NMR (CDCl_3_, 500 MHz), δ (ppm): 1.39 (t, *J* = 7.0 Hz, 3H, H11), 4.37 (q, *J* = 7.0 Hz, 2H, H10), 6.93 (t, *J* = 7.5 Hz, 1H, H5), 7.49 (d, *J* = 8.0 Hz, 2H, H15, H17), 7.66 (d, *J* = 7.5 Hz, 1H, H6), 7.69 (s, 1H, H2), 7.75 (d, *J* = 8.5 Hz, 2H, H14, H18), 9.98 (d, *J* = 7.0 Hz, 1H, H4). ^13^C NMR (CDCl_3_, 125 MHz), δ (ppm): 14.5 (C11), 61.3 (C10), 110.3 (C1), 112.4 (C7), 115.0 (C5), 122.0 (C3), 128.1 (C4), 128.9 (C15, C17), 129.7 (C2), 130.5 (C16, C18), 132.1 (C6), 135.8 (C8), 138.0 (C13), 138.3 (C16), 163.9 (C9), 184.1 (C12). Anal. Calcd. for C_18_H_13_BrClNO_3_: C, 53.16; H, 3.22; N, 3.44. Found: C, 53.15; H, 3.21; N, 3.46.

Ethyl ester of 8-bromo-3-(4-bromobenzoyl)indolizine-1-carboxylic acid **6b**. Cream solid, yield = 18%, m.p. = 155–156 °C. IR (KBr), ν (cm^−1^): 3092, 2963, 1737, 1705, 1622, 1520, 1352, 1261, 1168, 1089, 1031, 799. ^1^H NMR (CDCl_3_, 500 MHz), δ (ppm): 1.39 (t, *J* = 7.0 Hz, 3H, H11), 4.37 (q, *J* = 7.0 Hz, 2H, H10), 6.93 (t, *J* = 7.5 Hz, 1H, H5), 7.65–7.68 (overlapping signals, 5H, H6, H15, H17, H14, H18), 7.69 (s, 1H, H2), 9.99 (dd, *J* = 7.0; 0.5 Hz, 1H, H4). ^13^C NMR (CDCl_3_, 125 MHz), δ (ppm): 14.5 (C11), 61.3 (C10), 110.1 (C1), 112.4 (C7), 115.1 (C5), 122.0 (C3), 126.7 (C16), 128.1 (C4), 129.7 (C2), 130.7 (C14, C18), 131.9 (C15, C17), 132.1 (C6), 135.8 (C8), 138.5 (C13), 163.9 (C9), 184.2 (C12). Anal. Calcd. for C_18_H_13_Br_2_NO_3_: C, 47.92; H, 2.90; N, 3.10. Found: C, 47.91; H, 2.87; N, 3.11.

Ethyl ester of 8-bromo-3-(4-cyanobenzoyl)indolizine-1-carboxylic acid **6c**. Yellow solid, yield = 18%, m.p. = 154–155 °C. IR (KBr), ν (cm^−1^): 3092, 2992, 2224, 1730, 1624, 1523, 1471, 1429, 1356, 1217, 1170, 769. ^1^H NMR (CDCl_3_, 500 MHz), δ (ppm): 1.39 (t, *J* = 7.0 Hz, 3H, H11), 4.37 (q, *J* = 7.0 Hz, 2H, H10), 6.98 (t, *J* = 7.5 Hz, 1H, H5), 7.64 (s, 1H, H2), 7.71 (dd, *J* = 7.5; 1.0 Hz, 1H, H6), 7.82 (d, *J* = 8.5 Hz, 2H, H15, H17), 7.88 (d, *J* = 8.5 Hz, 2H, H14, H18), 10.02 (dd, *J* = 7.0; 0.5 Hz, 1H, H4). ^13^C NMR (CDCl_3_, 125 MHz), δ (ppm): 14.5 (C11), 61.4 (C10), 110.9 (C1), 112.5 (C7), 115.3 (C16), 115.5 (C5), 118.2 (CN), 121.6 (C3), 128.2 (C4), 129.5 (C14, C18), 129.9 (C2), 132.5 (C15, C17), 132.6 (C6), 136.2 (C8), 143.5 (C13), 163.8 (C9), 183.3 (C12). Anal. Calcd. for C_19_H_13_BrN_2_O_3_: C, 57.45; H, 3.30; N, 7.05. Found: C, 57.45; H, 3.28; N, 7.06.

Ethyl ester of 8-bromo-3-(4-methoxybenzoyl)indolizine-1-carboxylic acid **6d**. Yellow solid, yield = 15%, m.p. = 154–155 °C. IR (KBr), ν (cm^−1^): 3080, 2975, 1737, 1623, 1605, 1470, 1427, 1354, 1222, 1161, 1090, 757. ^1^H NMR (CDCl_3_, 500 MHz), δ (ppm): 1.38 (t, *J* = 7.0 Hz, 3H, H11), 3.90 (s, 1H, OCH_3_), 4.37 (q, *J* = 7.0 Hz, 2H, H10), 6.88 (t, *J* = 7.5 Hz, 1H, H5), 7.01 (d, *J* = 8.5 Hz, 2H, H15, H17), 7.61 (d, *J* = 7.5 Hz, 1H, H6), 7.73 (s, 1H, H2), 7.83 (d, *J* = 8.5 Hz, 2H, H14, H18), 9.93 (d, *J* = 7.0 Hz, 1H, H4). ^13^C-NMR (CDCl_3_, 125 MHz), δ (ppm): 14.5 (C11), 55.6 (OMe), 61.1 (C10), 109.7 (C1), 112.2 (C7), 113.9 (C15, C17), 114.5 (C5), 122.4 (C3), 127.9 (C4), 129.2 (C2), 131.4 (C6), 131.5 (C14, C18), 132.1 (C13), 135.3 (C8), 162.8 (C16), 164.1 (C9), 184.4 (C12). Anal. Calcd. for C_19_H_16_BrNO_4_: C, 56.73; H, 4.01; N, 3.48. Found: C, 56.71; H, 4.00; N, 3.49.

Ethyl ester of 8-bromo-3-(3,4-dimethoxybenzoyl)indolizine-1-carboxylic acid **6f**. Brown solid, yield = 20%, m.p. = 190–192 °C. IR (KBr), ν (cm^−1^): 3092, 2992, 2224, 1730, 1624, 1523, 1471, 1429, 1356, 1217, 1170, 769. ^1^H NMR (CDCl_3_, 500 MHz), δ (ppm): 1.39 (t, *J* = 7.0 Hz, 3H, H11), 3.96 (s, 3H, H19), 3.98 (s, 3H, H20), 4.37 (q, *J* = 7.0 Hz, 2H, H10), 6.89 (t, *J* = 7.5 Hz, 1H, H5), 6.97 (d, *J* = 8.0 Hz, 1H, H17), 7.42 (d, *J* = 2.0 Hz, 1H, H14), 7.47 (dd, *J* = 8.5; 3.5 Hz, 1H, H18), 7.62 (dd, *J* = 7.5; 1.0 Hz, 1H, H6), 7.77 (s, 1H, H2), 9.90 (dd, *J* = 7.0; 0.5 Hz, 1H, H4). ^13^C NMR (CDCl_3_, 125 MHz), δ (ppm): 14.5 (C11), 56.2 (C19), 56.3 (C20), 61.1 (C10), 109.8 (C1), 110.2 (C17), 111.2 (C14), 112.3 (C7), 114.6 (C5), 122.5 (C3), 123.8 (C18), 127.9 (C4), 129.3 (C2), 131.6 (C6), 132.3 (C13), 135.4 (C8), 149.2 (C15), 152.6 (C16), 164.1 (C9), 184.4 (C12). Anal. Calcd. for C_20_H_18_BrNO_5_: C, 55.57; H, 4.20; N, 3.24. Found: C, 55.57; H, 4.18; N, 3.25.

Diethyl ester of 3-(4-chlorobenzoyl)indolizine-1,7-dicarboxylic acid **7a**. White-yellowish solid, yield = 64%, m.p. = 179–180 °C. IR (KBr), ν (cm^−1^): 3133, 2986, 1719, 1622, 1528, 1469, 1352, 1286, 1251, 1215, 764. ^1^H NMR (CDCl_3_, 500 MHz), δ (ppm): 1.43 (t, *J* = 7.0 Hz, 3H, H11), 1.44 (t, *J* = 7.5 Hz, 3H, H21), 4.41 (q, *J* = 7.0 Hz, 2H, H10), 4.45 (q, *J* = 7.0, 2H, H20), 9.05 (bs, 1H, H7), 9.88 (d, *J* = 7.0 Hz, 1H, H4). ^13^C NMR (CDCl_3_, 125 MHz), δ (ppm): 14.4 (C11), 14.6 (C21), 60.7 (C10), 62.0 (C20), 109.3 (C1), 114.5 (C5), 121.8 (C7), 123.4 (C3), 128.6 (C6), 128.8 (C4), 128.9 (C2), 129.0 (C15, C17), 130.6 (C14, C18), 137.8 (C13), 138.4 (C16), 138.6 (C8), 163.6 (C9), 164.8 (C19), 184.6 (C12). Anal. Calcd. for C_21_H_18_ClNO_5_: C, 63.08; H, 4.54; N, 3.50. Found: C, 63.07; H, 4.53; N, 3.52.

Diethyl ester of 3-(4-bromobenzoyl)indolizine-1,7-dicarboxylic acid **7b**. Yellow solid, yield = 68%, m.p. = 175–176 °C. IR (KBr), ν (cm^−1^): 3132, 2984, 1718, 1622, 1528, 1469, 1285, 1250, 1215, 724. ^1^H NMR (CDCl_3_, 500 MHz), δ (ppm): 1.43 (t, *J* = 7.0 Hz, 3H, H11), 1.45 (t, *J* = 7.5 Hz, 3H, H21), 4.40 (q, *J* = 7.0 Hz, 2H, H10), 4.45 (q, *J* = 7.5 Hz, 2H, H20), 7.63 (dd, *J* = 7.0; 1.5 Hz, 1H, H5), 7.67 (d, *J* = 8.5 Hz, 2H, H15, H17), 7.71 (d, *J* = 8.5 Hz, 2H, H14, H18), 7.82 (s, 1H, H2), 9.06 (bs, 1H, H7), 9.89 (d, *J* = 7.0 Hz, 1H, H4). ^13^C NMR (CDCl_3_, 125 MHz), δ (ppm): 14.4 (C11), 14.6 (C21), 60.7 (C10), 62.0 (C20), 109.4 (C1), 114.5 (C5), 121.8 (C7), 123.3 (C3), 126.9 (C16), 128.6 (C4), 128.9 (C6), 129.0 (C2), 130.7 (C14, C18), 131.9 (C15, C17), 138.3 (C13), 138.6 (C8), 163.6 (C9), 164.8 (C19), 184.7 (C12). Anal. Calcd. for C_21_H_18_BrNO_5_: C, 56.77; H, 4.08; N, 3.15. Found: C, 56.77; H, 4.06; N, 3.16.

Diethyl ester of 3-(4-cyanobenzoyl)indolizine-1,7-dicarboxylic acid **7c**. Yellow solid, yield = 62%, m.p. = 180–181 °C. IR (KBr), ν (cm^−1^): 3128, 2988, 2230, 1716, 1621, 1530, 1470, 1353, 1288, 1251, 1221, 764. ^1^H NMR (CDCl_3_, 500 MHz), δ (ppm): 1.42 (t, *J* = 7.0 Hz, 3H, H11), 1.45 (t, *J* = 7.0 Hz, 3H, H21), 4.40 (q, *J* = 7.0 Hz, 2H, H10), 4.45 (q, *J* = 7.0 Hz, 2H, H20), 7.66 (dd, *J* = 7.0; 1.5 Hz, 1H, H5), 7.77 (s, 1H, H2), 7.83 (d, J = 8.5 Hz, 2H, H15, H17), 7.90 (d, J = 8.5 Hz, 2H, H14, H18), 9.05 (d, *J* = 1.0 Hz, 1H, H7), 9.91 (dd, *J* = 7.5; 0.5 Hz, 1H, H4). ^13^C NMR (CDCl_3_, 125 MHz), δ (ppm): 14.4 (C11), 14.6 (C21), 60.8 (C10), 62.1 (C20), 109.8 (C1), 114.9 (C5), 115.3 (C16), 118.1 (C22), 121.8 (C7), 122.9 (C3), 128.7 (C4), 129.3 (C2), 129.4 (C6), 129.5 (C14, C18), 132.5 (C15, C17), 138.9 (C8), 143.2 (C13), 163.4 (C9), 164.6 (C19), 183.8 (C12). HRMS (ESI, positive mode *m*/*z* calcd for C_22_H_18_N_2_O_5_ [M^+^]: 390.1216; Found: 390.1209. Anal. Calcd. for C_22_H_18_N_2_O_5_: C, 67.69; H, 4.65; N, 7.18. Found: C, 67.70; H, 4.63; N, 7.19.

Diethyl ester of 3-(4-methoxybenzoyl)indolizine-1,7-dicarboxylic acid **7d**. Yellow solid, yield = 60%, m.p. = 136–137 °C. IR (KBr), ν (cm^−1^): 3135, 2981, 1713, 1630, 1603, 1525, 1472, 1284, 1258, 1200, 1028, 763. ^1^H NMR (CDCl_3_, 500 MHz), δ (ppm): 1.41–1.45 (overlapping signals, 6H, H11, H21), 3.90 (s, 3H, OMe), 4.41 (q, *J* = 7.0 Hz, 2H, H10), 4.44 (q, *J* = 7.0 Hz, 2H, H20), 7.01 (d, *J* = 8.5 Hz, 2H, H15, H17), 7.58 (dd, *J* = 7.0; 1.5 Hz, 1H, H5), 7.85 (d, *J* = 8.5 Hz, 2H, H14, H18), 7.86 (s, 1H, H2), 9.03 (dd, *J* = 1.5; 0.5 Hz, 1H, H7), 9.83 (dd, *J* = 7.5; 0.5 Hz, 1H, H4). ^13^C NMR (CDCl_3_, 125 MHz), δ (ppm): 14.4 (C11), 14.6 (C21), 55.6 (OMe), 60.5 (C10), 61.9 (C20), 108.9 (C1), 113.9 (C15, C17), 114.0 (C5), 121.8 (C7), 123.9 (C3), 128.2 (C4, C2), 128.5 (C6), 131.5 (C14, C18), 131.9 (C13), 138.1 (C8), 163.0 (C16), 163.9 (C9), 164.9 (C19), 184.9 (C12). Anal. Calcd. for C_22_H_21_NO_6_: C, 66.83; H, 5.35; N, 3.54. Found: C, 66.82; H, 5.33; N, 3.55.

Diethyl ester of 3-(3,4,5-trimethoxybenzoyl)indolizine-1,7-dicarboxylic acid **7e**. Yellow solid, yield = 40%, m.p. = 166–167 °C. IR (KBr), ν (cm^−1^): 2947, 1716, 1705, 1637, 1618, 1471, 1385, 1356, 1208, 1133, 767. ^1^H NMR (CDCl_3_, 500 MHz), δ (ppm): 1.41–1.46 (overlapping signals, 6H, H11, H21), 3.92 (s, 6H, 2 × OMe), 3.96 (s, 3H, OMe), 4.41–4.46 (overlapping signals, 4H, H10, H20), 7.09 (s, 2H, H14, H18), 7.62 (dd, *J* = 7.0; 1.5 Hz, 1H, H5), 7.92 (s, 1H, H2), 9.06 (bs, 1H, H7), 9.85 (dd, *J* = 7.5; 0.5 Hz, 1H, H4). ^13^C NMR (CDCl_3_, 125 MHz), δ (ppm): 14.4 (C11), 14.6 (C21), 56.5 (2 × OMe), 61.2 (OMe), 60.6 (C10), 62.0 (C20), 106.8 (C14, C18), 109.2 (C1), 114.3 (C5), 121.9 (C7), 123.8 (C3), 128.6 (C4, C6), 128.9 (C2), 134.6 (C13), 138.4 (C8), 141.7 (C16), 153.2 (C15, C17), 163.7 (C9), 164.9 (C19), 185.1 (C12). Anal. Calcd. for C_24_H_25_NO_8_: C, 63.29; H, 5.53; N, 3.08. Found: C, 63.27; H, 5.52; N, 3.10.

Diethyl ester of 3-(3,4-dimethoxybenzoyl)indolizine-1,7-dicarboxylic acid **7f**.

Yellow solid, yield = 55%, m.p. = 168–169 °C. IR (KBr), ν (cm^−1^): 3135, 2981, 1713, 1630, 1603, 1525, 1472, 1284, 1258, 1200, 1028, 763. ^1^H NMR (CDCl_3_, 500 MHz), δ (ppm): 1.42–1.47 (overlapping signals, 6H, H11, H21), 3.97 (s, 3H, H22), 3.99 (s, 3H, H23), 4.41 (q, *J* = 7.0 Hz, 2H, H10), 4.45 (q, *J* = 7.0 Hz, 2H, H20), 6.98 (d, *J* = 8.5 Hz, 1H, H17), 7.45 (d, *J* = 2.0 Hz, 1H, H14), 7.50 (dd, *J* = 8.0; 2.0 Hz, 1H, H18), 7.61 (dd, *J* = 7.0; 2.0 Hz, 1H, H5), 7.91 (s, 1H, H2), 9.06 (d, *J* = 0.5 Hz, 1H, H7), 9.83 (d, *J* = 7.5 Hz, 1H, H4). ^13^C NMR (CDCl_3_, 125 MHz), δ (ppm): 14.4 (C11), 14.6 (C21), 56.2 (C22), 56.3 (C23), 60.6 (C10), 61.9 (C20), 109.0 (C1), 110.3 (C17), 111.8 (C18), 114.1 (C5), 121.9 (C7), 123.9 (C14, C3), 128.3 (C6), 128.5 (C4), 128.6 (C2), 132.1 (C13), 138.3 (C8), 149.3 (C15), 152.8 (C16), 163.9 (C9), 165.0 (C19), 184.8 (C12). Anal. Calcd. for C_23_H_23_NO_7_: C, 64.93; H, 5.45; N, 3.29. Found: C, 64.92; H, 5.44; N, 3.31.

Diethyl ester of 3-benzoylindolizine-1,7-dicarboxylic acid **7g**. Yellow solid, yield = 50%, m.p. = 109–110 °C. IR (KBr), ν (cm^−1^): 3139, 3059, 1721, 1625, 1530, 1474, 1352, 1228, 1207, 762. ^1^H NMR (CDCl_3_, 500 MHz), δ (ppm): 1.43 (t, *J* = 7.0 Hz, 3H, H11), 1.45 (t, *J* = 7.5 Hz, 3H, H21), 4.41 (q, *J* = 7.0 Hz, 2H, H10), 4.45 (q, *J* = 7.0 Hz, 2H, H20), 7.53 (t, *J* = 7.0 Hz, 2H, H15, H17), 7.59–7.64 (overlapping signals, 2H, H16, H5), 7.83 (dd, *J* = 8.0; 1.5 Hz, 2H, H14, H18), 7.87 (s, 1H, H2), 9.06 (dd, *J* = 1.5; 1.0 Hz, 1H, H7), 9.93 (dd, *J* = 7.5; 1.0 Hz, 1H, H4). ^13^C NMR (CDCl_3_, 125 MHz), δ (ppm): 14.4 (C11), 14.6 (C21), 60.6 (C10), 62.0 (C20), 109.2 (C1), 114.4 (C5), 121.9 (C7), 123.8 (C3), 128.6 (C15, C17, C4), 129.2 (C6, C14, C18), 129.3 (C2), 132.0 (C16), 138.5 (C8), 139.6 (C13), 163.8 (C9), 164.9 (C19), 186.1 (C12). Anal. Calcd. for C_21_H_19_NO_5_: C, 69.03; H, 5.24; N, 3.83. Found: C, 69.03; H, 5.22; N, 3.84.

#### 3.1.4. X-Ray Crystallography

Single-crystal X-ray diffraction data were collected using an Oxford-Diffraction XCALIBUR Eos CCD or XtaLAB Synergy, Dualflex, HyPix diffractometers. The unit cell determination and data integration were carried out using the CrysAlisPro package from Oxford Diffraction [37]. Multi-scan correction for absorption was applied. The structure was solved with SHELXT program using the intrinsic phasing method and refined by the full-matrix least-squares method on *F*^2^ with SHELXL [38,39]. Olex2 was used as an interface to the SHELX programs [40]. Non-hydrogen atoms were refined anisotropically. Hydrogen atoms were added in idealized positions and refined using a riding model. Selected crystallographic data and structure refinement details are provided in Tables S1 and S2 and the corresponding CIF-files in the Supplementary Material. The supplementary crystallographic data can be obtained free of charge via www.ccdc.cam.ac.uk/conts/retrieving.html (accessed on 30 July 2025) or from the Cambridge Crystallographic Data Centre, 12 Union Road, Cambridge CB2 1EZ, UK; fax: (+44) 1223-336-033; or deposit@ccdc.ca.ac.uk.

### 3.2. Biological Activity

#### 3.2.1. Cell Proliferation Assay

The NCI-60 cell line panel provides a comprehensive platform for evaluating the anticancer potential of new compounds, as it comprises 60 distinct human cancer cell lines representative of a wide range of malignancies, including renal, lung, colon, breast, leukemia, CNS, melanoma, ovarian and prostate cancers. This extent makes it a critical early-stage tool for drug discovery.

In our study, compounds were tested against the full NCI-60 panel at the National Cancer Institute (Rockville, MD, USA) using the sulforhodamine B (SRB) assay under a standard 48 h exposure protocol [28,29,30]. Cells were cultured in RPMI-1640 medium supplemented with 5% fetal bovine serum and 2 mM L-glutamine, then seeded into 96-well microtiter plates (100 µL/well) at densities of 5000–40,000 cells/well depending on doubling times. After 24 h of incubation at 37 °C in a humidified 5% CO_2_ atmosphere, two reference plates were fixed with trichloroacetic acid (TCA) to establish baseline cell counts (Tz).

Test compounds, prepared as frozen DMSO stock solutions at 400× the desired final concentration, were thawed and diluted in complete medium containing gentamicin prior to use. Serial dilutions were performed to obtain five graded concentrations plus control, and 100 µL of each dilution was added to the wells. Plates were then incubated for 48 h under the same conditions.

For adherent cells, the assay was terminated by gentle addition of cold TCA (final concentration 10%), followed by fixation at 4 °C for 1 h, washing, and staining with 0.4% SRB in 1% acetic acid. After washing and drying, bound dye was solubilized with 10 mM Tris base, and absorbance was read at 515 nm. For suspension cells, fixation was carried out with 16% TCA, but the subsequent steps remained identical.

Growth inhibition was quantified using absorbance values from time zero (Tz), control growth (C), and treated cells (Ti at five concentrations). Percentage growth inhibition (PGI) was calculated as follows:

PGI = [(Ti − Tz)/(C − Tz)] × 100, for Ti ≥ Tz

PGI = [(Ti − Tz)/Tz] × 100, for Ti < Tz

At the level of individual experiments all endpoint values will have a count of 1 and a standard deviation of 0.

#### 3.2.2. Molecular Docking

The binding to αβ-tubulin of synthesized indolizine derivatives was investigated through molecular docking simulations, which were performed with AutoDock 4.2.6 [41] employing the Lamarckian Genetic Algorithm (LGA) [42]. The receptor model was generated based on the X-ray structure of the tubulin–colchicine complex available in the Protein Data Bank (PDB ID: 4O2B) [31]. In this model, the crystallographic GDP and GTP nucleotides were retained along with Ca^2+^ and Mg^2+^ ions, while all water molecules, colchicine and any other co-crystallized ligands or organic solvent molecules were removed. During the docking, the tubulin backbone and side chains were treated as rigid.

All ligands (phenstatin, colchicine, and synthetized ligands **5c**, **6c**, and **7g**) were built using GaussView 5.0.9 [43] and geometry-optimized at the PM6 semiempirical level with Gaussian 16 [44]. A 60 × 60 × 60 points cubic grid box with a distance of 0.375 Å was generated, centered on the geometric center of the site occupied by colchicine in the crystallographic complex (coordinates: x = 17.019, y = 65.993, z = 43.390), ensuring that the pocket was fully enclosed. For each ligand, 2000 independent LGA runs were performed with a population size of 300, while all other genetic algorithm parameters were maintained at default values. Docking results were clustered and ranked according to predicted binding free energy. The most favorable binding configuration for every ligand was further analyzed, with 3D visualizations created in PyMOL [45] and 2D interaction schemes produced with Maestro [46].

#### 3.2.3. In Silico ADME and Toxicity Predictions

In silico ADME evaluation of the most active compounds (**5c**, **6c** and **7g**) was conducted using the SwissADME web tool (http://swissadme.ch/index.php accessed on 15 July 2025), assessing its molecular properties, pharmacokinetics, drug-likeness, and medicinal chemistry profile [34]. Additionally, the compound’s toxicological profile was predicted using the Cell-Line Cytotoxicity Predictor (CLC-Pred) web service (https://www.way2drug.com/clc-pred/ accessed on 20 July 2025), which estimates cytotoxicity across a panel of 391 tumor cell lines and 47 normal human cell lines derived from various tissues [35,36].

## 4. Conclusions

In summary, two series of bromo-substituted indolizines (6-bromo-substituted indolizines **5a**–**f** and 8-bromo-substituted indolizines **6a**–**f**) and one series of 1,7-dicarboxyethyl disubstituted indolizines **7a**–**g** were designed and synthesized using the corresponding pyridinium ylides as key intermediates. Somewhat unanticipated, we obtained two types of bromo-substituted indolizines from the 3+2 cycloaddition reaction of 3-bromopyridinium ylides to ethyl propiolate, which correspond to the two possible 1,3-dipole structures that can be adopted by that ylides. The structures of all new compounds were confirmed by elemental and spectral analysis and X-ray diffraction in case of indolizine **6a**. Twelve intermediate pyridinium salts and twelve indolizinic compounds were evaluated in vitro for their anticancer effects on NCI’s panel of 60 cancer cell lines, with three compounds **5c**, **6c** and **7g** showing notable growth inhibition of several cell lines (including non-small lung cancer, glioblastoma, melanoma and RXF-393 renal cancer cell lines). HOP-62 non-small cell lung and SNB-75 glioblastoma cells exhibited particularly sensitivity also to the cytotoxic properties of **5c**, **6c** and **7g** compounds compared to the other cell lines. Compound **5c** showed also cytotoxic effect against lung cancer cells NCI-H226, melanoma cells SK-MEL-2 and renal cancer cells RXF-393, being the most potent from the tested compounds in terms and cytostatic and cytotoxic properties. Molecular docking analysis revealed that active compounds **5c**, **6c**, and **7g** demonstrated good binding affinities, as evidenced by favorable docking scores, and formed stable interactions with colchicine site of tubulin, suggesting a possible anticancer mechanism. The calculated ADMET parameters further indicated that these derivatives possess favorable physicochemical properties, promising oral bioavailability and drug-likeness, and minimal toxicity risks. Together, these findings highlight significant potential, especially in case compound **5c**, for the development of novel multifunctionalized indolizines with improved anticancer activity and pharmacokinetic profile.

## Figures and Tables

**Table 1:** Selected results of the in vitro growth inhibition (GI%) of tested compounds against human cancer cell lines in the single-dose assay ^a^.

Cell Type	Compound → Cell Line ↓	GI (%) (10^–5^ M) ^a^						
		1c	5a	5c	6a	6c	7d	7g
Leukemia	HL-60(TB)	26	32	38	**62**	18	49	36
	K-562	**61**	22	25	24	40	0	0
	MOLT-4	0	21	1	**53**	9	**54**	36
	RPMI-8226	38	27	42	30	25	11	0
	EKVX	9	11	**50**	9	27	6	19
Non-small Cell Lung Cancer	HOP-62	**54**	**55**	**100 ^b ^** **(34)**	**78**	**97**	21	**100 ^b ^** **(15)**
	HOP-92	0	20	**78**	0	**71**	6	35
	NCI-H226	31	16	**100 ^b ^** **(14)**	19	**54**	8	47
	NCI-H23	10	16	36	17	31	9	**50**
	NCI-H522	6	21	**60**	19	**52**	**54**	44
Colon Cancer	HCT-116	11	21	29	23	21	4	41
	HCT-15	4	20	19	20	20	27	42
	HT29	7	41	31	43	28	0	13
CNS Cancer	SF-268	13	19	**59**	9	45	0	25
	SNB-19	11	13	**51**	3	31	10	35
	SNB-75	0	1	**100 ^b ^** **(16)**	46	**100 ^b ^** **(15)**	20	**64**
Melanoma	SK-MEL-2	18	0	**100 ^b^** ** (7)**	11	**80**	6	15
	SK-MEL-28	17	9	45	4	26	0	32
	SK-MEL-5	10	24	**50**	31	38	**59**	**68**
	UACC-257	14	0	**70**	0	26	6	3
	UACC-62	13	6	**50**	0	48	0	11
Ovarian Cancer	IGROV1	0	2	**58**	9	18	0	16
	OVCAR-3	15	0	47	6	36	33	**54**
	OVCAR-4	9	6	**67**	8	**60**	1	20
	OVCAR-8	8	21	**65**	7	**54**	6	16
	SK-OV-3	0	0	52	15	30	10	0
Renal cancer	A498	7	2	41	0	33	2	16
	RXF 393	3	29	**100 ^b^** ** (4)**	45	**57**	9	8
	TK-10	2	0	**52**	14	47	10	10
Prostate cancer	PC-3	14	17	44	1	32	0	8
Breast cancer	MCF7	14	5	46	18	35	13	**62**
	HS 578T	24	10	**68**	13	**68**	10	11

^a^ Data obtained from NCI’s in vitro 60 cell one dose screening at 10^−5^ M; ^b^ Cytotoxic effect; the percentage of cytotoxicity is shown in parentheses. The best values in terms of cytotoxicity and in terms of growth inhibition caused by the tested compounds are highlighted in red and bold, and bold, respectively.

**Table 2:** Docking Data: Binding Energies 3D Poses and 2D Interaction Diagrams.

Ligand	Lowest Binding Energy (kcal/mol)	Binding Conformation	2D Interaction Diagram
Colchicine	−10.08	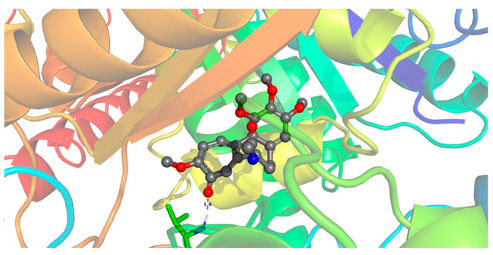	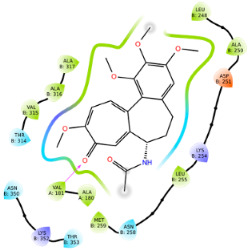
Phenstatin	−8.04	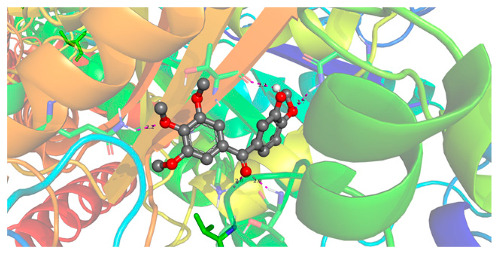	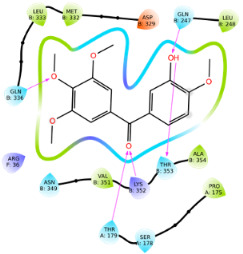
**5c**	−9.88	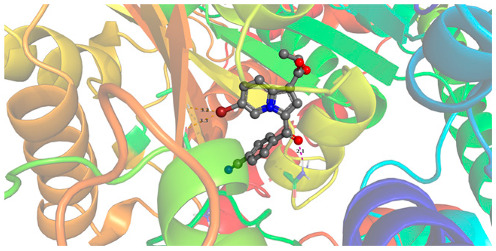	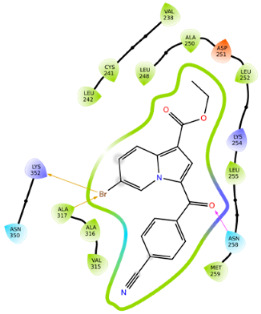
**6c**	−9.48	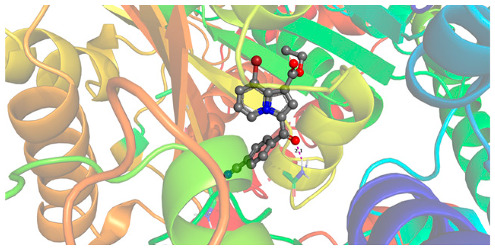	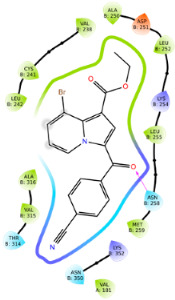
**7g**	−9.22	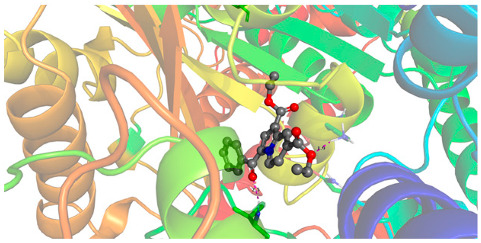	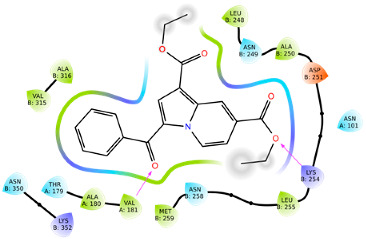

**Table 3:** In silico prediction of ADME parameters for compounds **5c**, **6c** and **7g**.

Compound → ADME Parameter ↓	5c	6c	7g
Physicochemical Properties			
Molecular weight	397.22 g/mol	397.22 g/mol	365.38 g/mol
Log *P*_o/w_ (MLOGP)	2.26	2.26	2.37
Number of H-bond acceptors	4	4	5
Number of H-bond donors	0	0	0
Number of rotatable bonds	5	5	8
TPSA	71.57 Å^2^	71.57 Å^2^	74.08 Å^2^
Pharmacokinetics			
Gastrointestinal (GI) absorption	high	high	high
Blood–brain barrier (BBB) permeant	yes	yes	yes
P-gp substrate	no	no	no
Drug likeness			
Log S (ESOL)	−5.44	−5.44	−4.89
Water solubility class	moderately soluble	moderately soluble	moderately soluble
Lipinski rule	no violation	no violation	no violation
Veber rule	no violation	no violation	no violation
Bioavailability	0.55	0.55	0.55
Medicinal Chemistry			
PAINS alerts	0	0	0
Brenk alerts	0	0	1: more_than_2_ esters
Synthetic accessibility	2.67	2.74	2.82

**Table 4:** Predicted cytotoxicity profiles of compounds **5c**, **6c**, and **7g** generated using the CLC-Pred web tool [36].

Compound	Pa	Pi	Cell-Line	Tissue/Organ	Type	IAP *
**5c**	0.578	0.002	SNU-398	Liver	Carcinoma	0.970
	0.530	0.016	SNB-75	CNS	Glioblastoma	0.877
**6c**	0.545	0.003	SNU-398	Liver	Carcinoma	0.970
	0.510	0.013	SNB-75	CNS	Glioblastoma	0.877
**7g**	0.578	0.002	SNU-398	Liver	Carcinoma	0.970
	0.574	0.012	SNB-75	CNS	Glioblastoma	0.877

* IAP (Invariant Accuracy of Prediction) is the average accuracy of prediction.

## Data Availability

The data are presented in the article/Supplementary Material. Any inquiry can be directed to the corresponding author.

## Supplementary Materials

The following supporting information can be downloaded at https://www.mdpi.com/article/10.3390/ijms26178368/s1.
