# Supplementary material for: Functionalized Indolizines as Potential Anticancer Agents: Synthetic, Biological and In Silico Investigations
Source: Int J Mol Sci. 2025 Aug 28;26(17):8368. doi: 10.3390/ijms26178368 (PMC12429123; doi:10.3390/ijms26178368)
Supplement: Supplementary file 1 [file ijms-26-08368-s001.zip › ijms-3822239-supplementary.pdf]

Supplementary material

for

Functionalized indolizines as potential anticancer agents: synthetic, biological and *in silico* investigations

Roxana Ciorteanu[1,2] Catalina Ionica Ciobanu[3], Narcis Cibotariu[4], Sergiu Shova[5], Vasilichia Antoci[2], Ionel I. Mangalagiu[2], Ramona Danac[2,*]

[1] "Alexandru Ioan Cuza" University of Iasi, ICI RECENT AIR Center, 11 Carol I, Iasi 700506, Romania; roxana.ciorteanu@uaic.ro (R.C)

[2] Faculty of Chemistry, Alexandru Ioan Cuza University of Iasi, 11 Carol I, Iasi 700506, Romania; roxana.ciorteanu@uaic.ro (R.C); vasilichia.antoci@uaic.ro (V.A.); ionelm@uaic.ro (I.I.M.); rdanac@uaic.ro (R.D.)

[3] Institute of Interdisciplinary Research-CERNESIM Centre, Alexandru Ioan Cuza University of Iasi, 11 Carol I, Iasi 700506, Romania; catalina.ciobanu@uaic.ro (C.I.C)

[4] Centre of Advanced Research in Bionanoconjugates and Biopolymers, "Petru Poni" Institute of Macromolecular Chemistry of Romanian Academy, 41A Grigore Ghica Voda Alley, Iasi 700487, Romania; cibotariu.narcis@icmpp.ro (N.C.)

[5] Department of Inorganic Polymers, "Petru Poni" Institute of Macromolecular Chemistry of Romanian Academy, 41A Grigore Ghica Voda Alley,Iasi 700487, Romania; shova@icmpp.ro (S.S.)

[*] Correspondence: rdanac@uaic.ro (R.D.)

Table of Contents

Figure S1. ^{1}H-NMR spectrum of compound **1c**……………………………………..…………..…...…….3

Figure S2. ^{1}C-NMR spectrum of compound **1c**………………………………………………..…..…… 3

Figure S3. ^{1}H-NMR spectrum of compound **5a**…………………………...............…………...….4

Figure S4. ^{1}C-NMR spectrum of compound **5a**………………………………………….…………...4

Figure S5. ^{1}H-NMR spectrum of compound **5c**…………………………………………….……....…5

Figure S6. ^{1}C-NMR spectrum of compound **5c**…………………………………….……….…..….5

Figure S7. ^{1}H-NMR spectrum of compound **6a** ………………………….……………………..….6

Figure S8. ^{1}C-NMR spectrum of compound **6a** ……………..………………………………………… 6

Figure S9. ^{1}H-NMR spectrum of compound **6c**………..………..…………………………………7

Figure S10. ^{1}C-NMR spectrum of compound **6c…**……………………………………….….……7

Figure S11. ^{1}H-NMR spectrum of compound **7d**……………………………………….………..8

Figure S12. ^{1}C-NMR spectrum of compound **7d…**…………………………………….…….…8

Figure S13. ^{1}H-NMR spectrum of compound **7g**……………………………………….…......9

Figure S14. ^{1}C-NMR spectrum of compound **7g** ……………………………………………..9

Figure S15. Results of the *in vitro* growth of cancer cell lines in the single-dose assay for compound **1c**….**10**

Figure S16. Results of the *in vitro* growth of cancer cell lines in the single-dose assay for compound **5a**….**11**

Figure S17. Results of the *in vitro* growth of cancer cell lines in the single-dose assay for compound **5c**…**12**

Figure S18. Results of the *in vitro* growth of cancer cell lines in the single-dose assay for compound **6a**…**13**

Figure S19. Results of the *in vitro* growth of cancer cell lines in the single-dose assay for compound **6c**….**14**

Figure S20. Results of the *in vitro* growth of cancer cell lines in the single-dose assay for compound **7d**….**15**

Figure S21. Results of the *in vitro* growth of cancer cell lines in the single-dose assay for compound **7g**….**16**

Table S1. Crystallographic data, details of data collection and structure refinement parameters for **6a**…......**17**

Table S2. Bond distances (Å) and angles (°) for **6a**……………………………………..……………......**18**

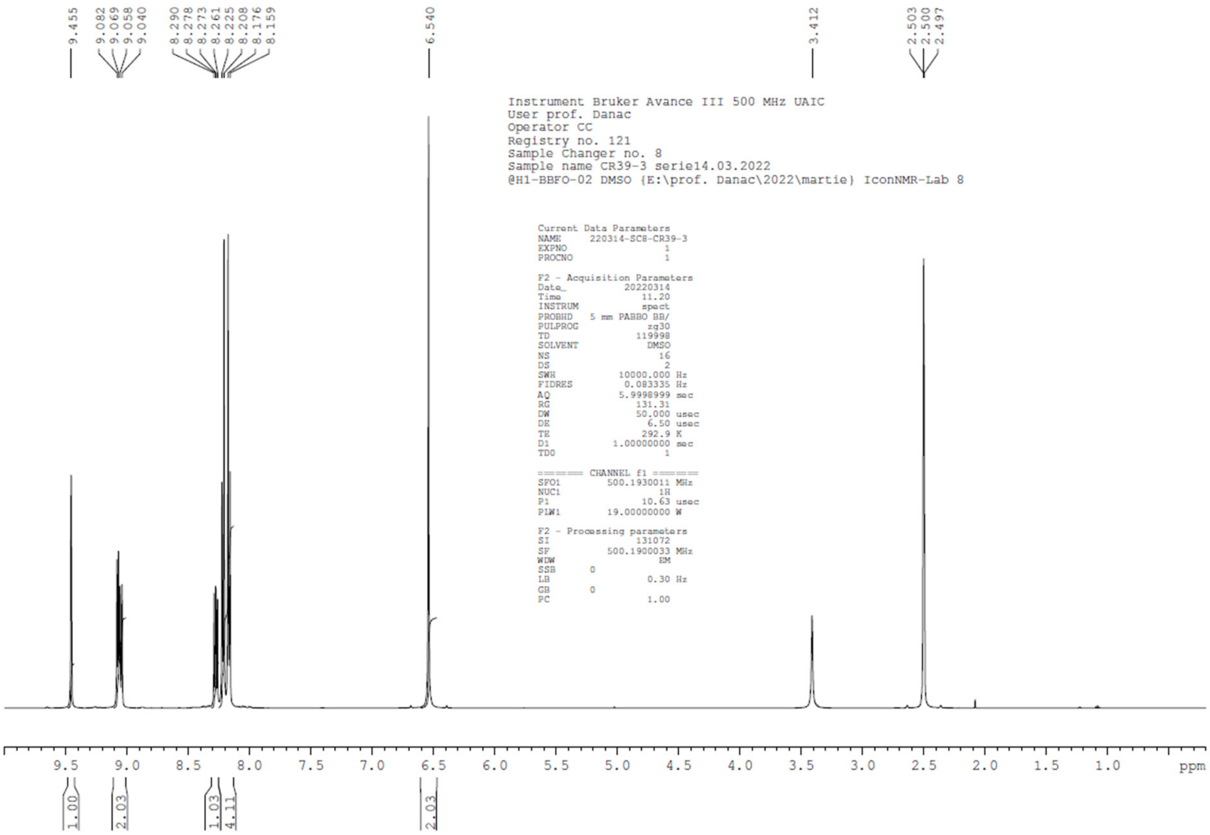

Figure S1. ^{1}H-NMR spectrum of compound **1c**

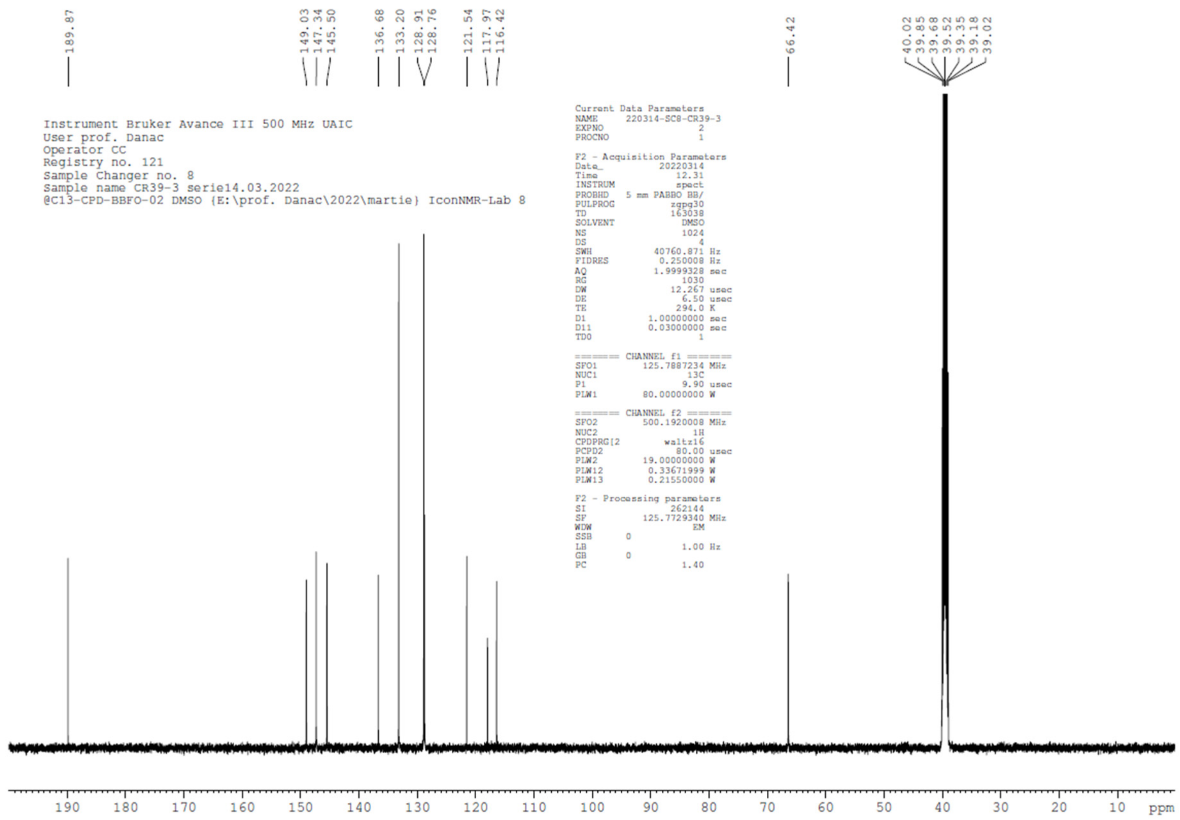

Figure S2. ^{1}C-NMR spectrum of compound **1c**

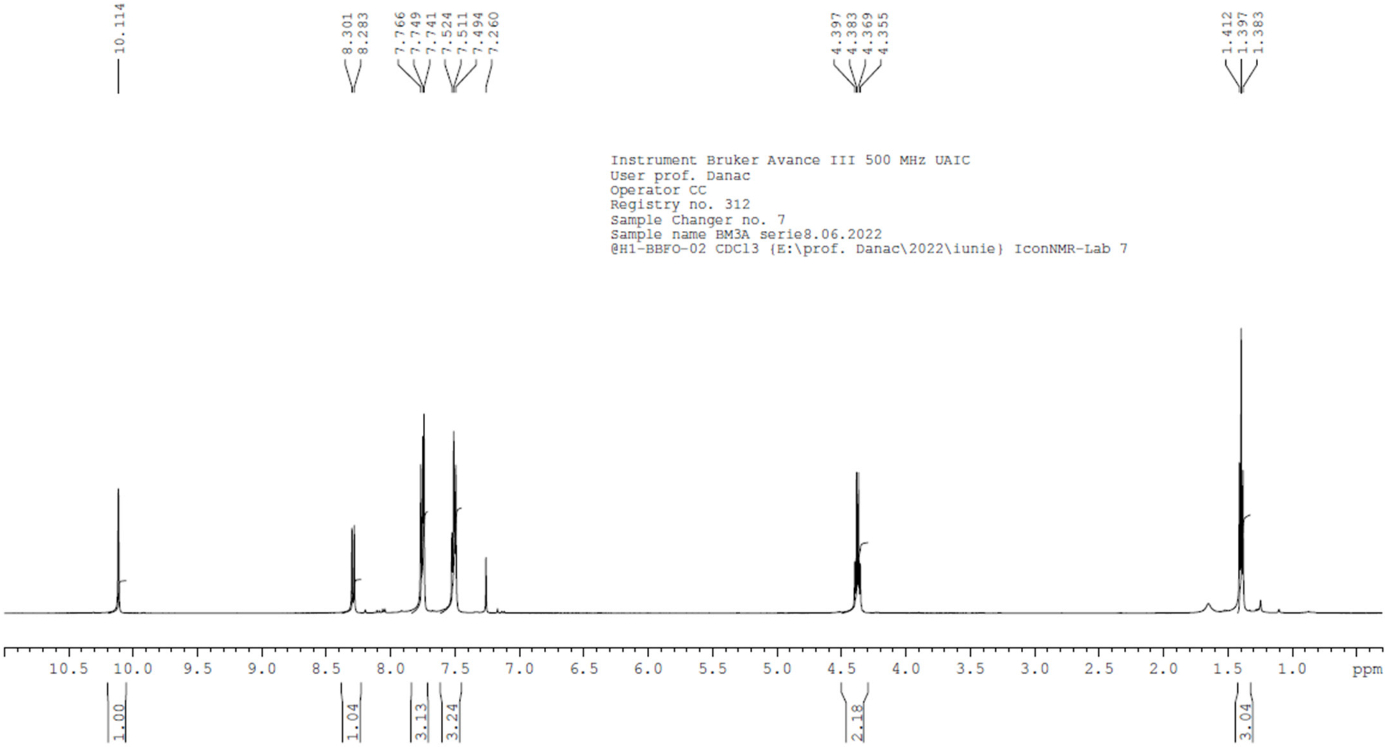

Figure S3. ^{1}H-NMR spectrum of compound **5a**

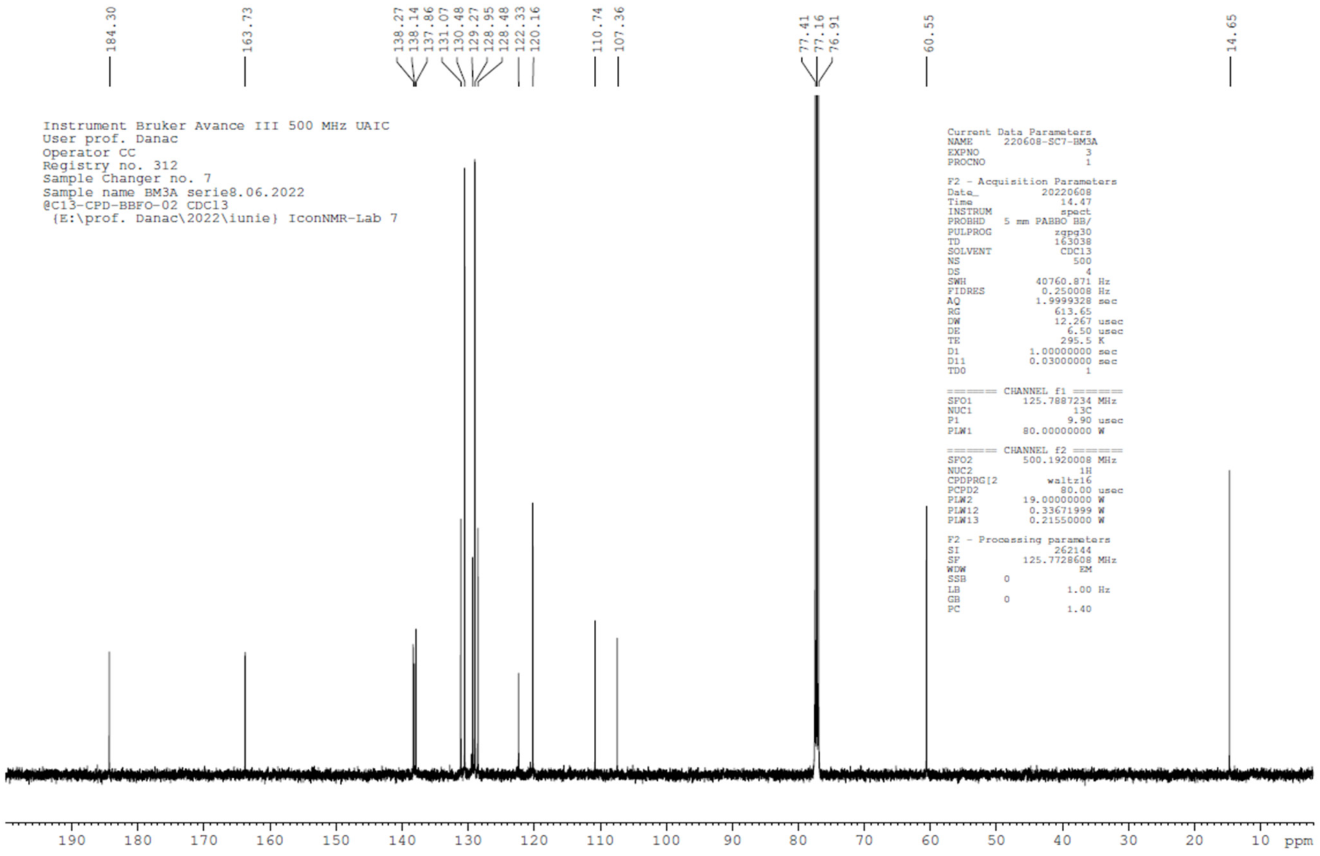

Figure S4. ^{1}C-NMR spectrum of compound **5a**

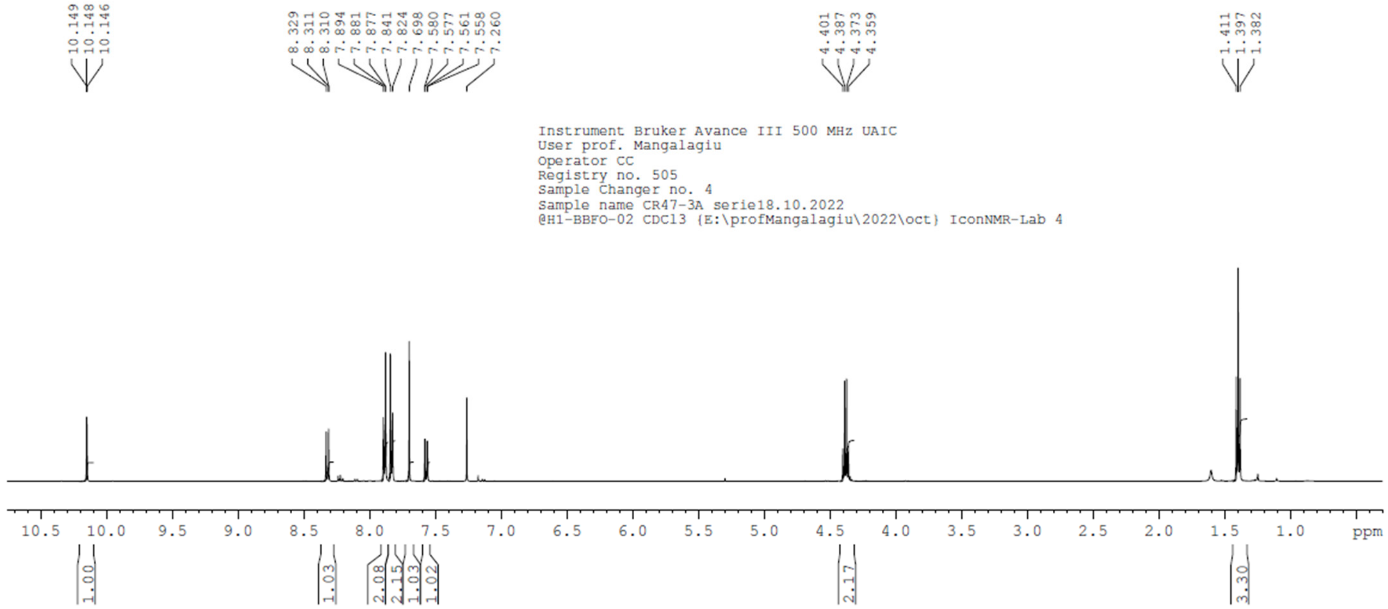

Figure S5. ^{1}H-NMR spectrum of compound **5c**

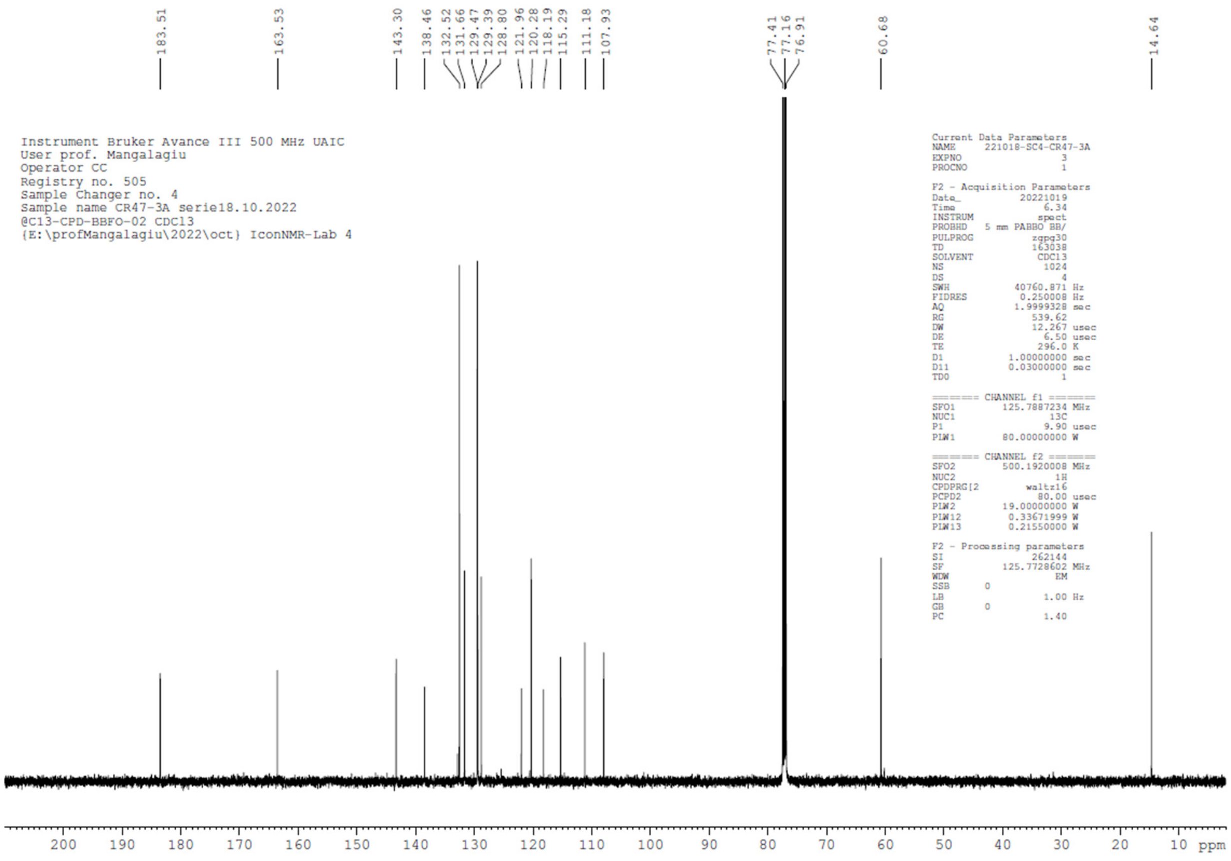

Figure S6. ^{1}C-NMR spectrum of compound **5c**

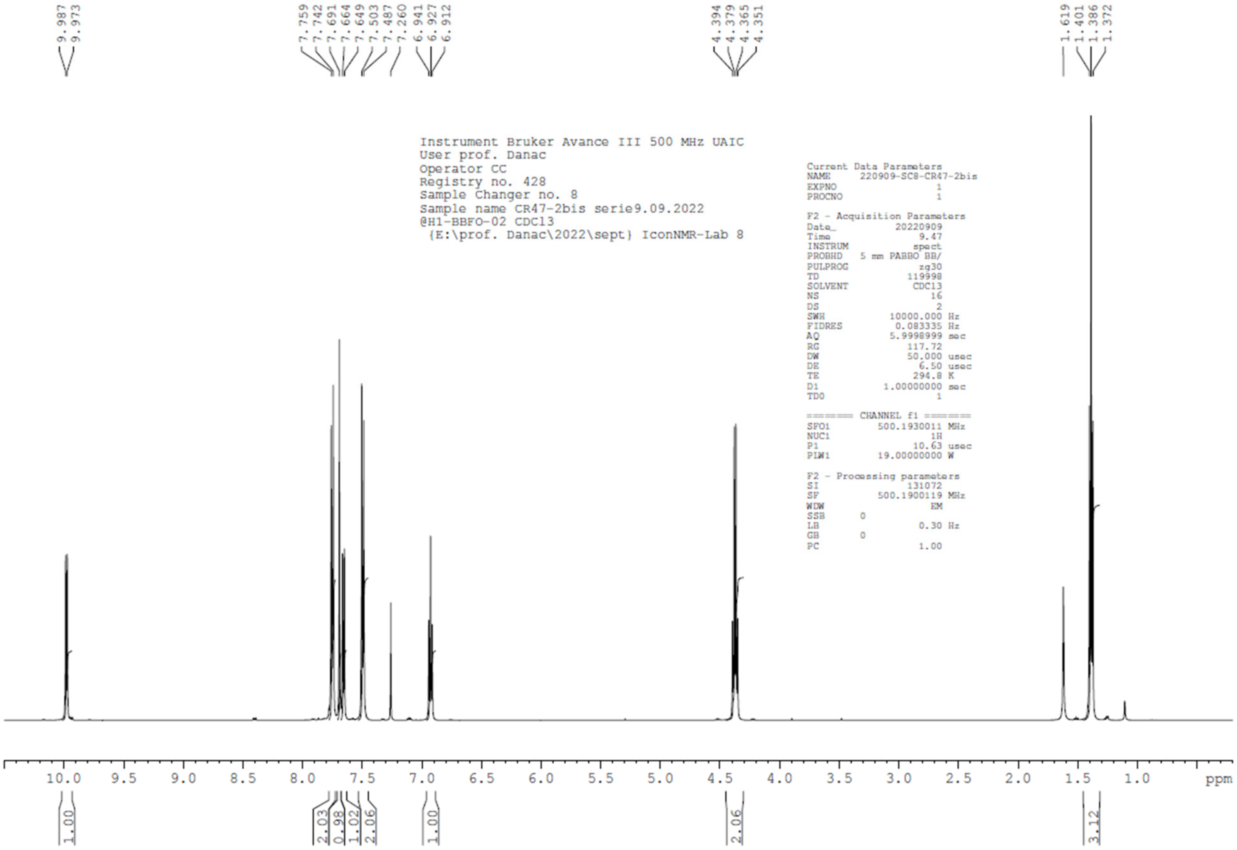

Figure S7. ^{1}H-NMR spectrum of compound **6a**

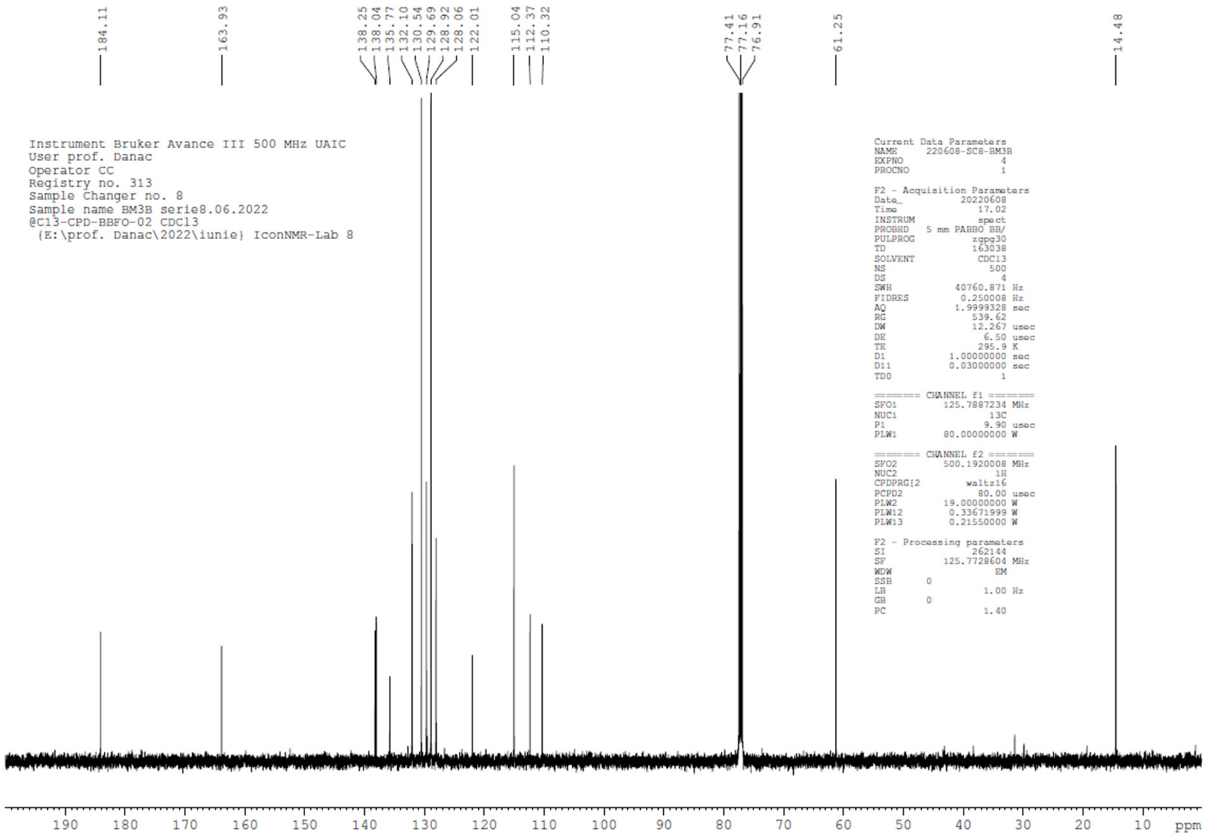

Figure S8. [1]C-NMR spectrum of compound **6a**

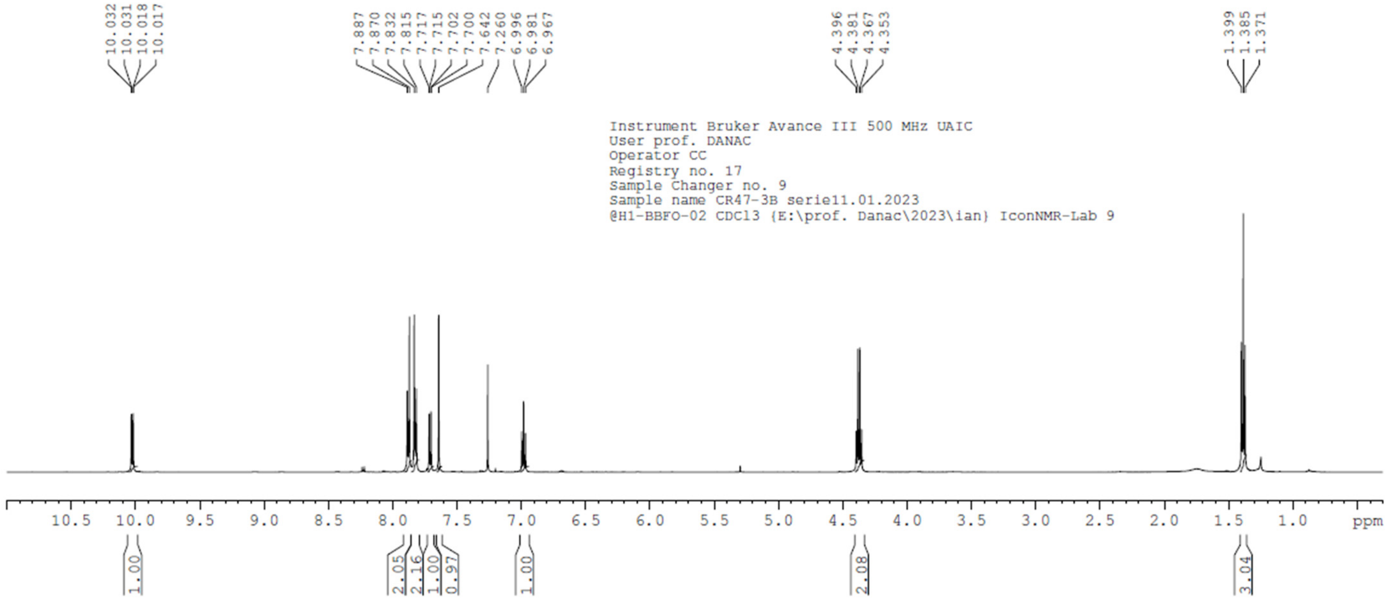

Figure S9. ^{1}H-NMR spectrum of compound **6c**

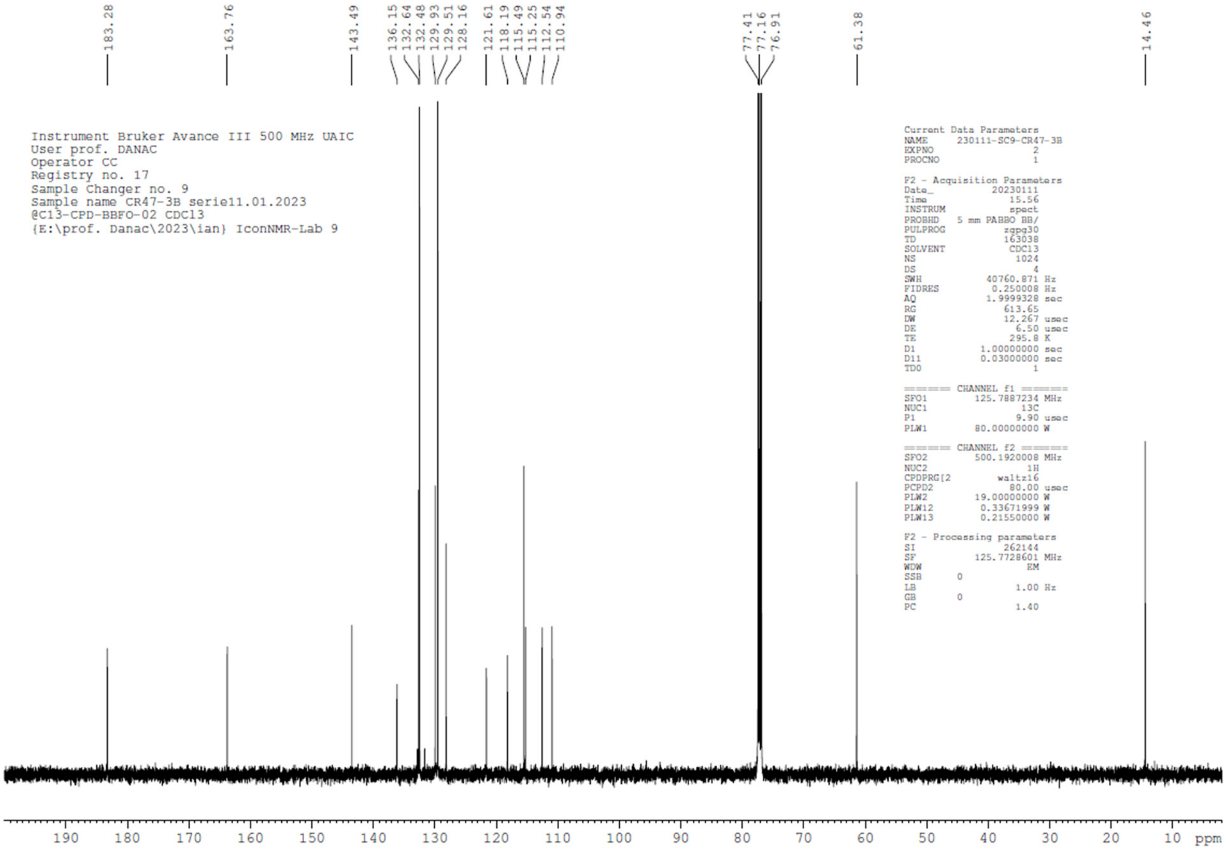

Figure S10. ^{1}C-NMR spectrum of compound **6c**

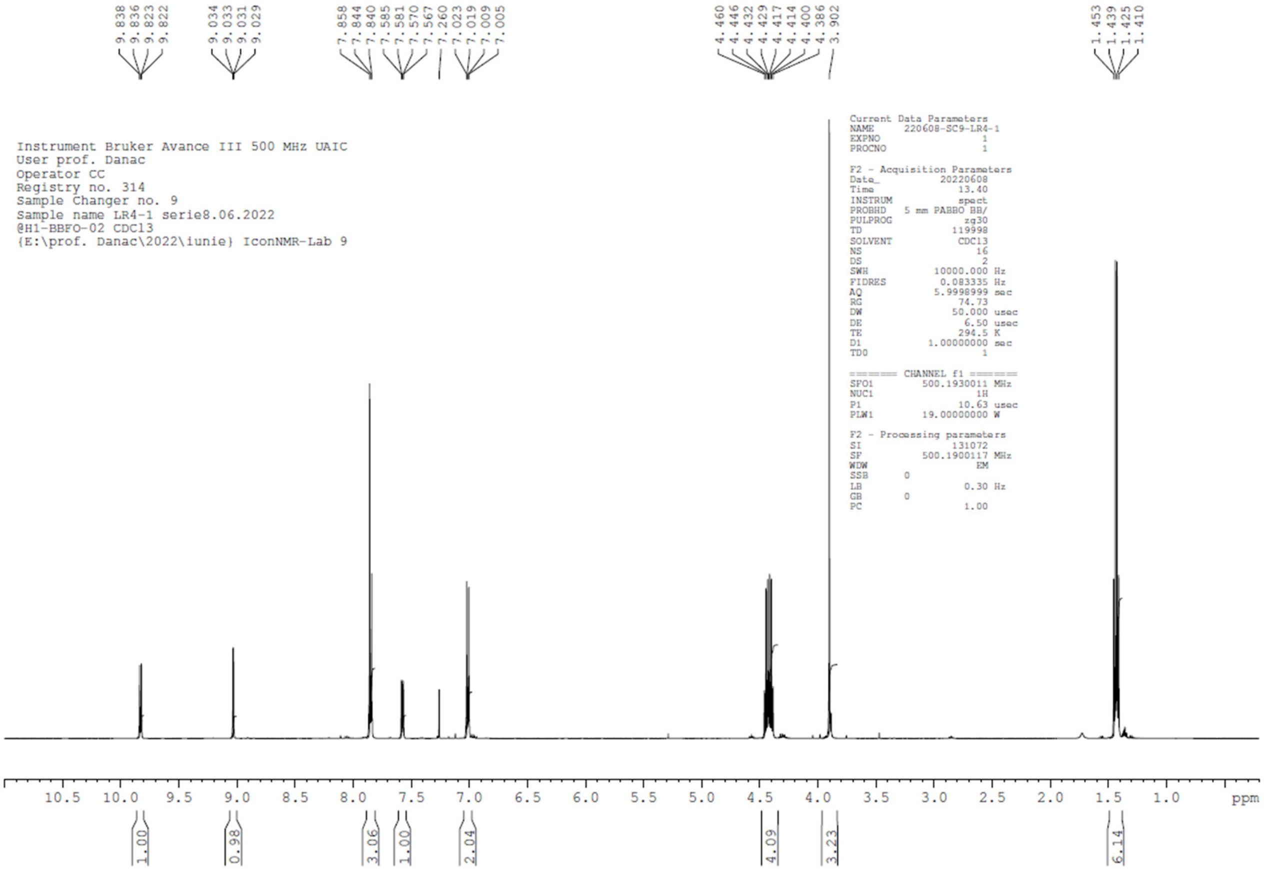

Figure S11. ^{1}H-NMR spectrum of compound **7d**

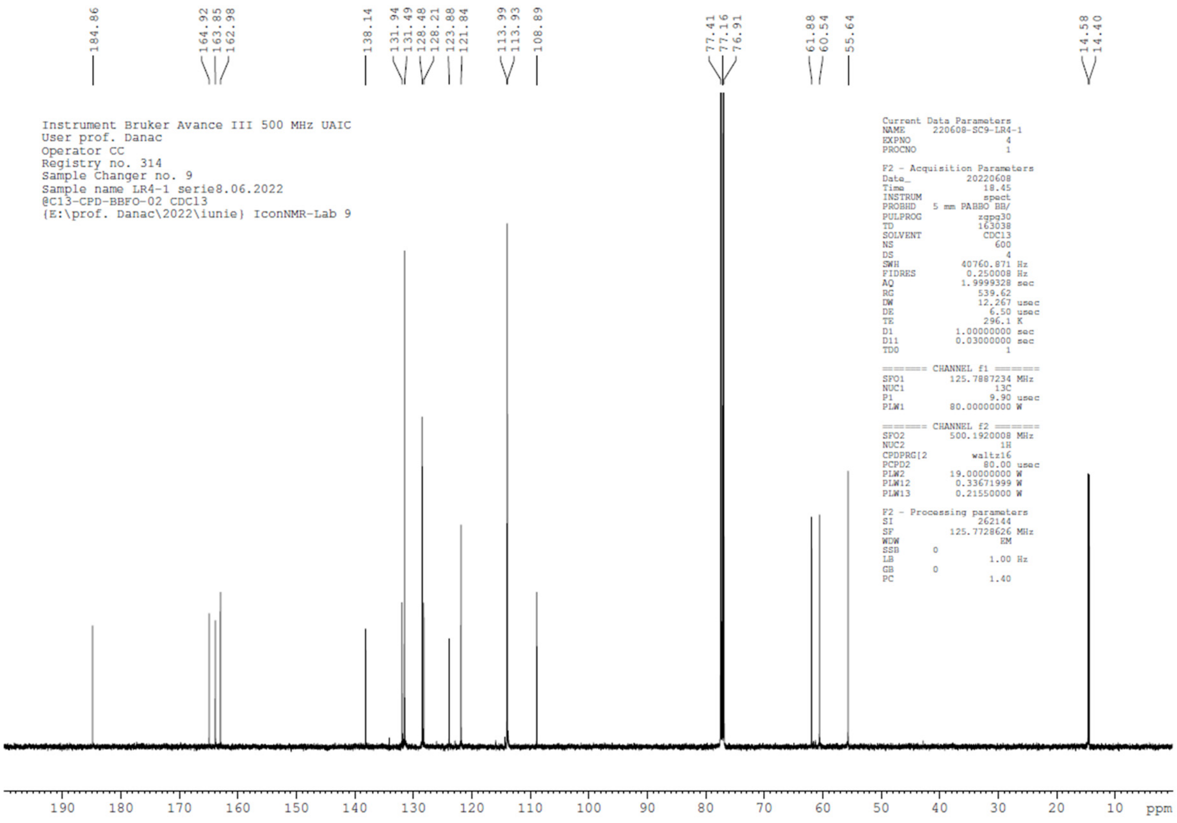

Figure S12. ^{1}C-NMR spectrum of compound **7d**

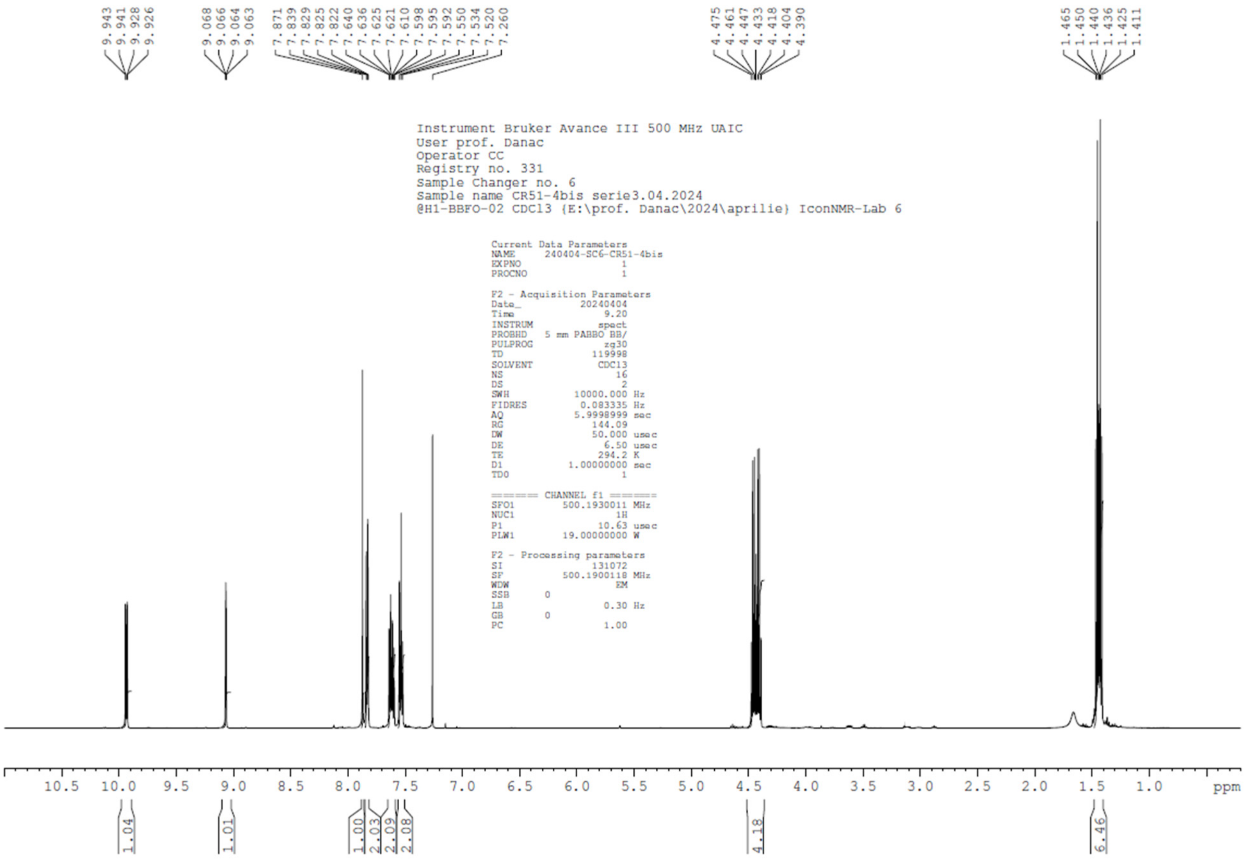

Figure S13. ^{1}H-NMR spectrum of compound **7g**

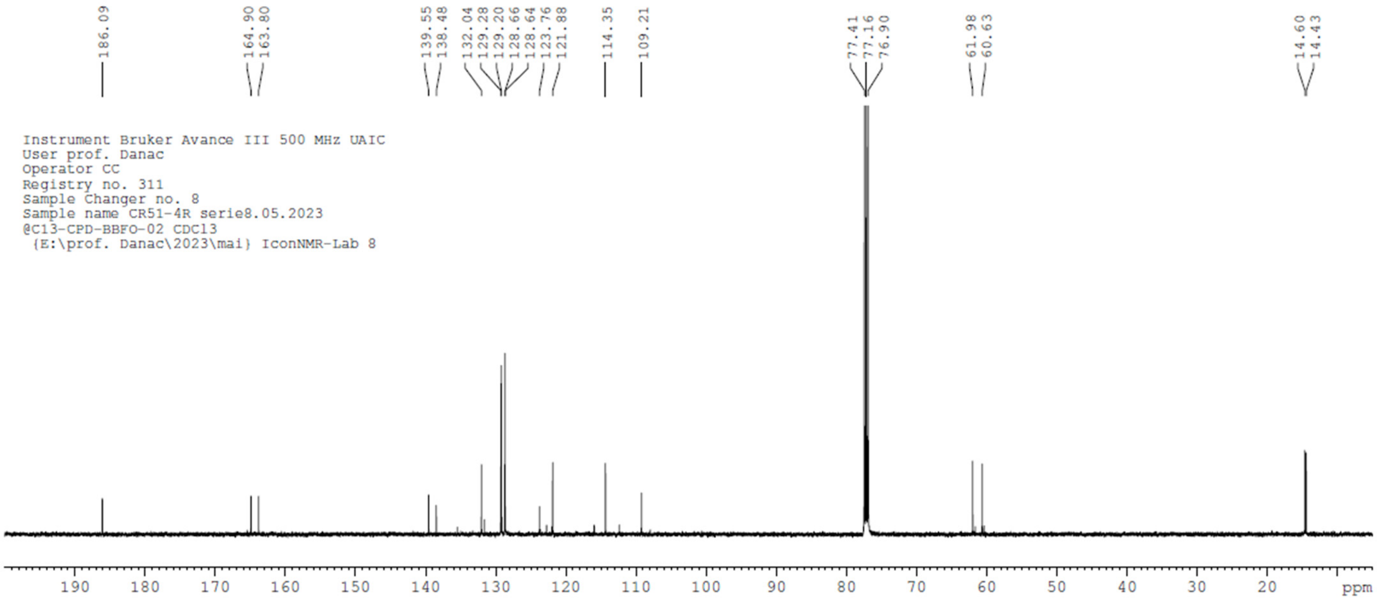

Figure S14. [1]C-NMR spectrum of compound **7g**

Developmental Therapeutics Program	NSC: D-850371 / 1	Conc: 1.000E-5 M	Test Date: Jan 8, 2024
One Dose Mean Graph	Experiment ID: 2401HT22		Report Date: Feb 22, 2024

Panel/Cell Line	Growth Percent
Leukemia	
CCRF-CEM	105.72
HL-60(TB)	73.92
K-562	38.83
MOLT-4	103.94
RPMI-8226	62.41
Non-Small Cell Lung Cancer	
A549/ATCC	74.60
EKVX	90.69
HOP-62	45.98
HOP-92	102.57
NCI-H226	68.39
NCI-H23	90.33
NCI-H322M	55.23
NCI-H460	95.83
NCI-H522	94.27
Colon Cancer	
COLO 205	86.57
HCC-2998	65.64
HCT-116	88.54
HCT-15	96.03
HT29	92.65
KM12	80.72
SW-620	96.97
CNS Cancer	
SF-268	88.68
SF-295	119.83
SF-539	103.38
SNB-19	89.17
SNB-75	101.96
U251	103.13
Melanoma	
LOX IMVI	111.19
MALME-3M	69.80
M14	83.31
MDA-MB-435	109.76
SK-MEL-2	82.33
SK-MEL-28	83.27
SK-MEL-5	91.01
UACC-257	85.74
UACC-62	87.43
Ovarian Cancer	
IGROV1	105.02
OVCAR-3	85.26
OVCAR-4	90.66
OVCAR-8	92.45
NCI/ADR-RES	99.00
SK-OV-3	100.97
Renal Cancer	
786-0	84.50
A498	93.37
ACHN	99.92
CAKI-1	105.19
RXF 393	96.92
SN12C	106.44
TK-10	97.61
Prostate Cancer	
PC-3	85.75
DU-145	96.78
Breast Cancer	
MCF7	86.45
HS 578T	75.62
BT-549	85.55
MDA-MB-468	63.48
Mean	88.56
Delta	49.73
Range	81.00

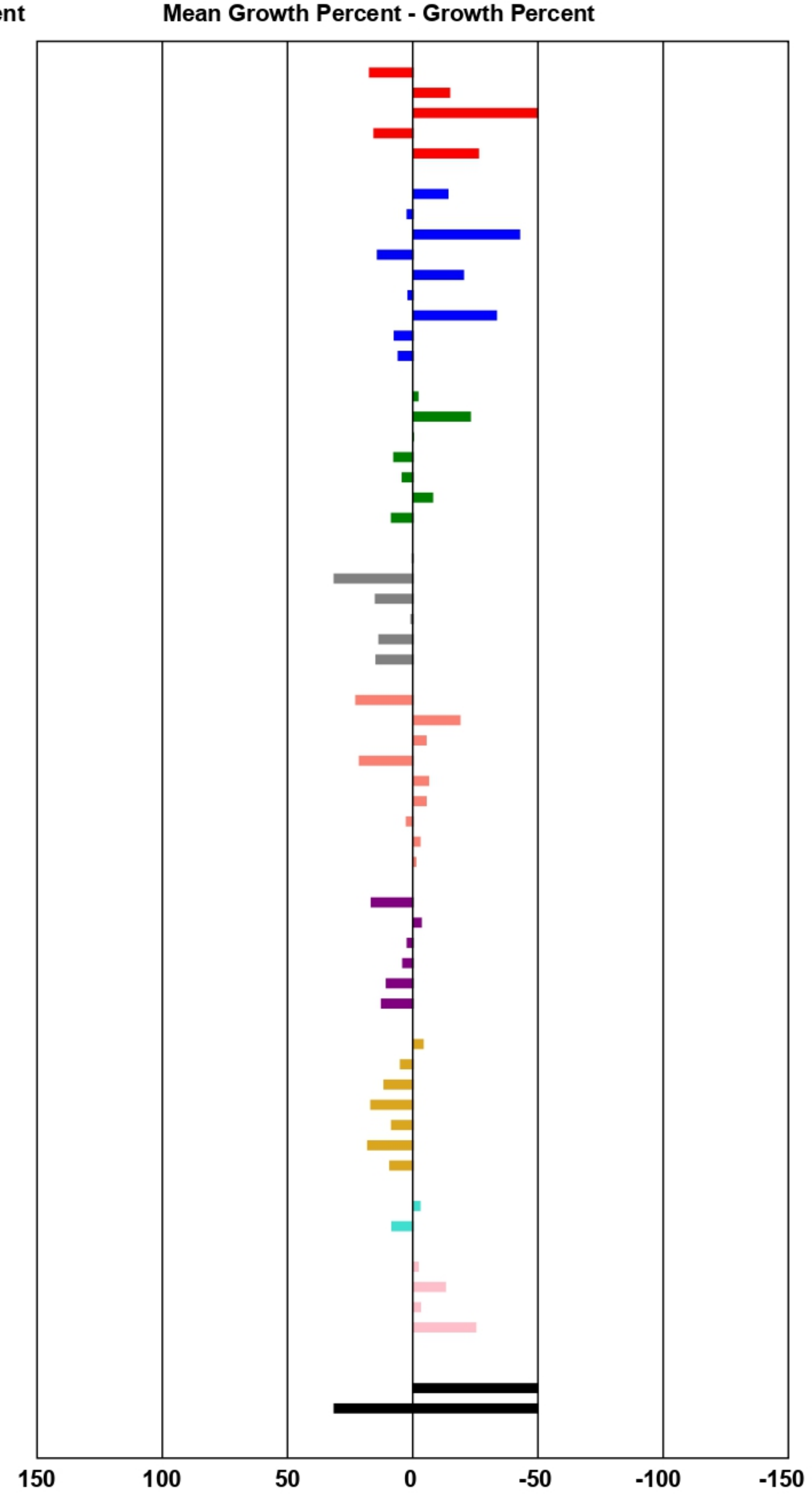

Figure S15. Results of the *in vitro* growth of cancer cell lines in the single-dose assay for compound **1c**

Developmental Therapeutics Program

One Dose Mean Graph

NSC: D-850642 / 1 | **Conc:** 1.000E-5 M | **Test Date:** Jan 8, 2024

Experiment ID: 2401HT23 | **Report Date:** Feb 22, 2024

Panel/Cell Line	Growth Percent
**Leukemia**	
CCRF-CEM	78.58
HL-60(TB)	68.05
K-562	78.14
MOLT-4	79.09
RPMI-8226	72.91
**Non-Small Cell Lung Cancer**	
A549/ATCC	82.29
EKVX	88.54
HOP-62	44.92
HOP-92	80.36
NCI-H226	83.98
NCI-H23	83.71
NCI-H322M	86.56
NCI-H460	80.24
NCI-H522	78.98
**Colon Cancer**	
COLO 205	72.57
HCC-2998	96.16
HCT-116	78.96
HCT-15	79.76
HT29	58.54
KM12	78.98
SW-620	86.49
**CNS Cancer**	
SF-268	81.46
SF-295	110.94
SF-539	95.86
SNB-19	88.68
SNB-75	98.76
U251	99.67
**Melanoma**	
LOX IMVI	97.25
MALME-3M	128.60
M14	106.92
MDA-MB-435	95.54
SK-MEL-2	102.66
SK-MEL-28	90.58
SK-MEL-5	76.53
UACC-257	99.91
UACC-62	94.22
**Ovarian Cancer**	
IGROV1	97.59
OVCAR-3	99.59
OVCAR-4	93.78
OVCAR-8	79.29
NCI/ADR-RES	98.56
SK-OV-3	107.23
**Renal Cancer**	
786-0	95.07
A498	98.44
ACHN	101.41
CAKI-1	115.94
RXF 393	71.36
SN12C	81.21
TK-10	104.66
**Prostate Cancer**	
PC-3	83.30
DU-145	94.26
**Breast Cancer**	
MCF7	94.74
HS 578T	90.31
BT-549	96.48
MDA-MB-468	92.85
Mean	89.12
Delta	44.20
Range	83.68

Mean Growth Percent - Growth Percent

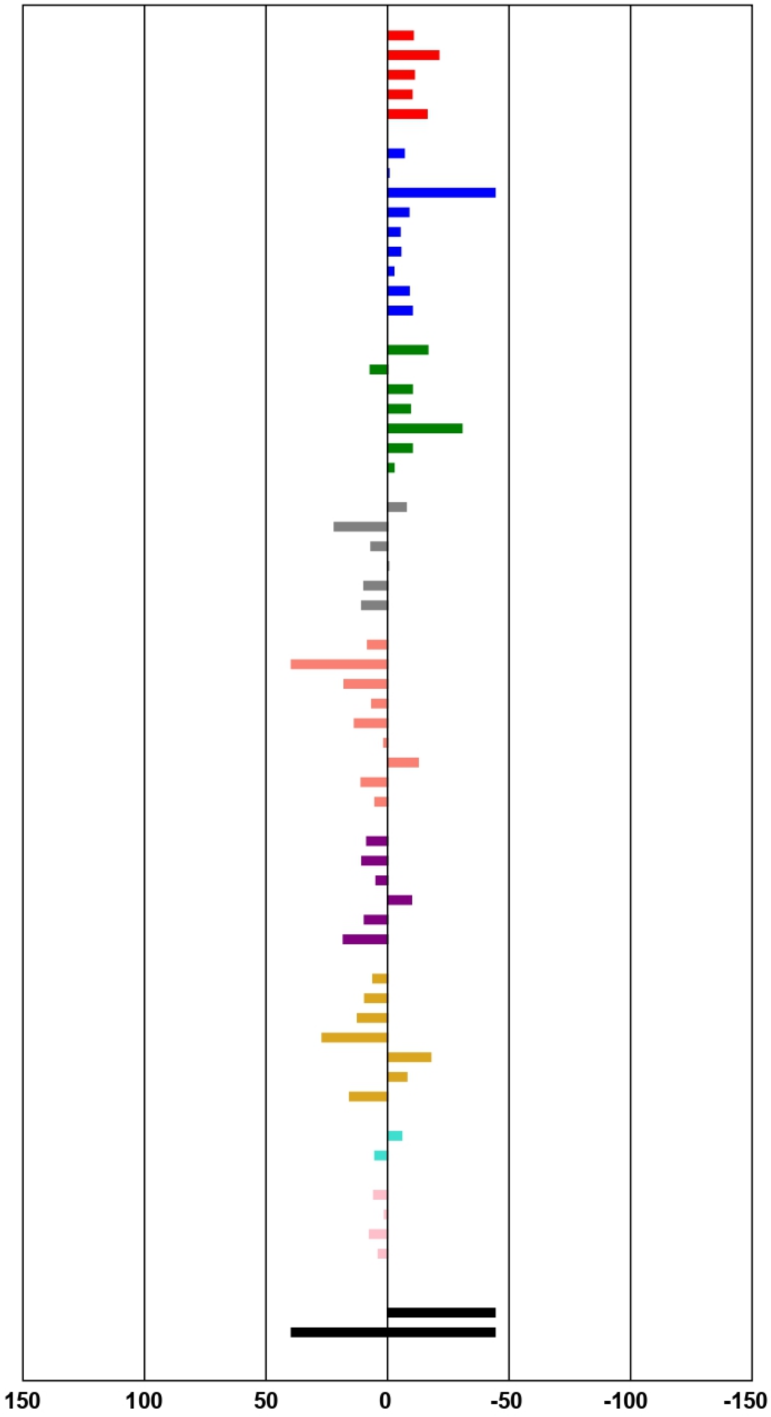

Figure S16. Results of the *in vitro* growth of cancer cell lines in the single-dose assay for compound **5a**

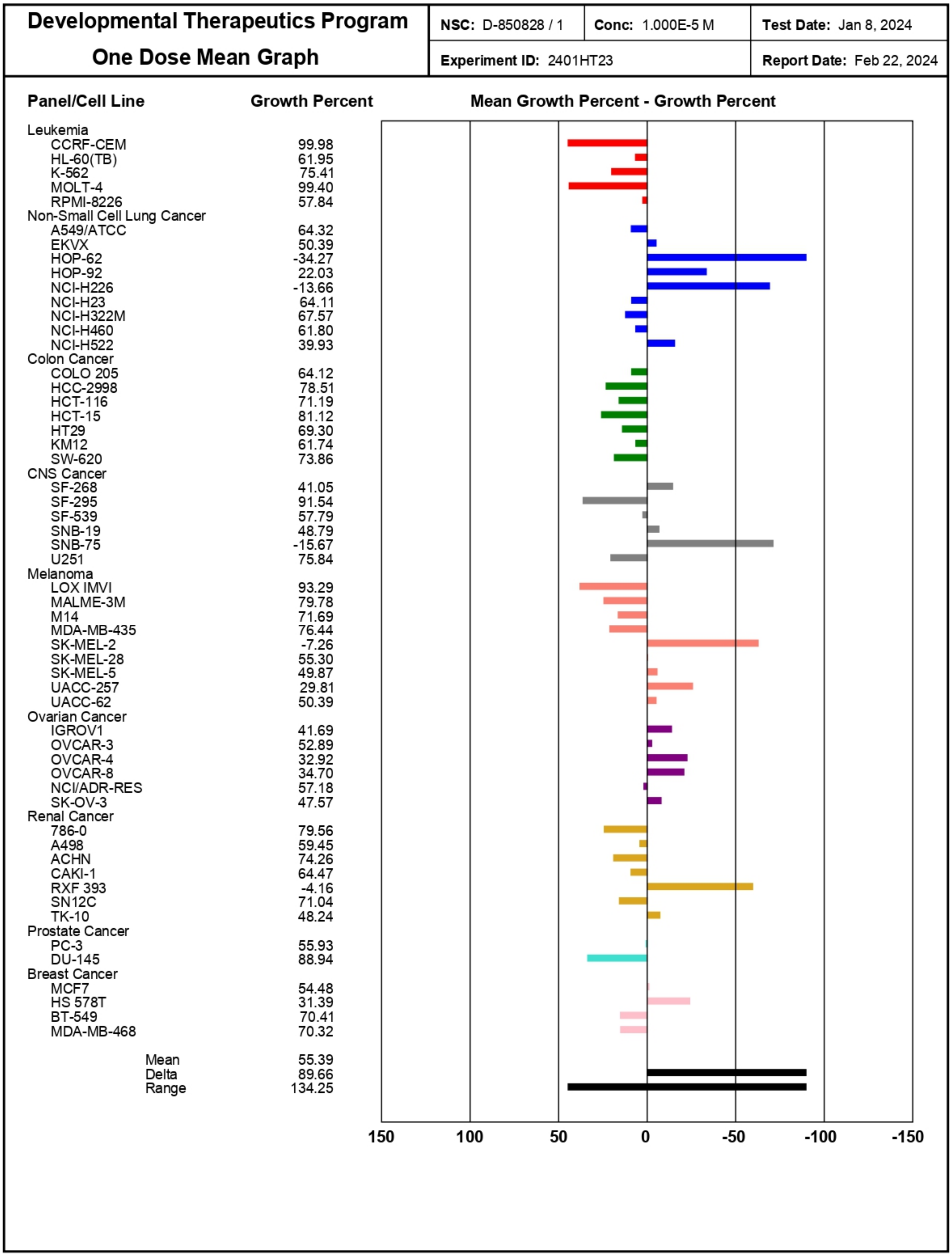

Figure S17. Results of the *in vitro* growth of cancer cell lines in the single-dose assay for compound **5c**

Developmental Therapeutics Program	NSC: D-850644 / 1	Conc: 1.000E-5 M	Test Date: Jan 8, 2024
One Dose Mean Graph	Experiment ID: 2401HT23		Report Date: Feb 22, 2024

Panel/Cell Line	Growth Percent
**Leukemia**	
CCRF-CEM	72.96
HL-60(TB)	37.71
K-562	76.39
MOLT-4	47.38
RPMI-8226	70.25
**Non-Small Cell Lung Cancer**	
A549/ATCC	89.25
EKVX	90.60
HOP-62	21.60
HOP-92	100.84
NCI-H226	81.25
NCI-H23	83.12
NCI-H322M	114.84
NCI-H460	90.31
NCI-H522	81.05
**Colon Cancer**	
COLO 205	90.74
HCC-2998	116.74
HCT-116	77.06
HCT-15	79.54
HT29	56.66
KM12	80.42
SW-620	92.72
**CNS Cancer**	
SF-268	90.76
SF-295	92.33
SF-539	91.35
SNB-19	96.60
SNB-75	54.21
U251	101.65
**Melanoma**	
LOX IMVI	71.55
MALME-3M	146.05
M14	116.96
MDA-MB-435	106.85
SK-MEL-2	88.78
SK-MEL-28	95.99
SK-MEL-5	69.21
UACC-257	122.61
UACC-62	106.25
**Ovarian Cancer**	
IGROV1	90.59
OVCAR-3	94.26
OVCAR-4	92.26
OVCAR-8	93.17
NCI/ADR-RES	99.17
SK-OV-3	84.90
**Renal Cancer**	
786-0	87.20
A498	100.68
ACHN	102.04
CAKI-1	116.56
RXF 393	55.39
SN12C	97.64
TK-10	86.43
**Prostate Cancer**	
PC-3	99.15
DU-145	93.03
**Breast Cancer**	
MCF7	81.92
HS 578T	86.88
BT-549	83.72
MDA-MB-468	47.33
Mean	87.18
Delta	65.58
Range	124.45

Mean Growth Percent - Growth Percent

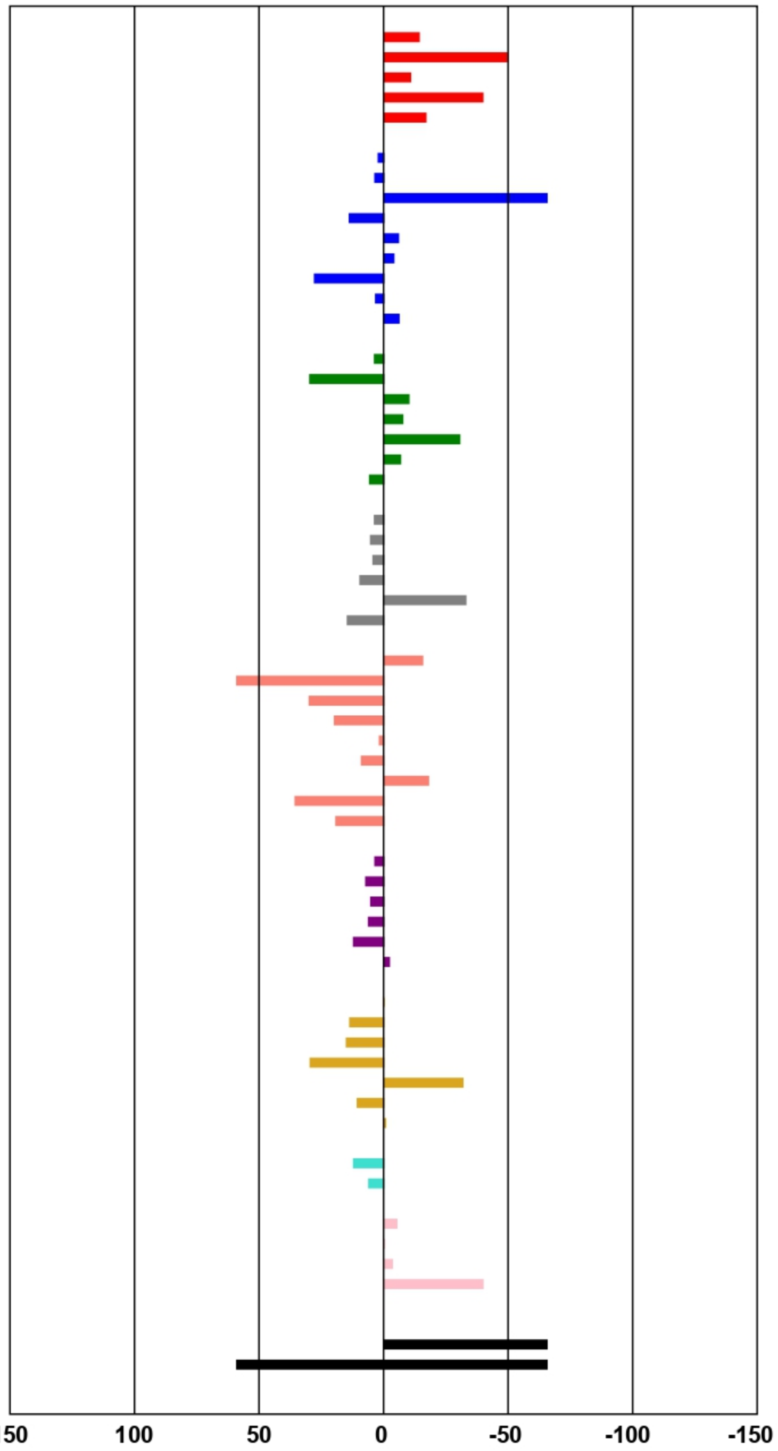

Figure S18. Results of the *in vitro* growth of cancer cell lines in the single-dose assay for compound **6a**

Developmental Therapeutics Program One Dose Mean Graph	NSC: D-850829 / 1	Conc: 1.000E-5 M	Test Date: Jan 8, 2024
	Experiment ID: 2401HT23		Report Date: Feb 22, 2024

Panel/Cell Line	Growth Percent
Leukemia	
CCRF-CEM	89.10
HL-60(TB)	81.81
K-562	60.13
MOLT-4	90.58
RPMI-8226	74.99
Non-Small Cell Lung Cancer	
A549/ATCC	78.96
EKVX	73.12
HOP-62	2.82
HOP-92	28.80
NCI-H226	46.37
NCI-H23	69.00
NCI-H322M	75.68
NCI-H460	66.72
NCI-H522	47.92
Colon Cancer	
COLO 205	78.79
HCC-2998	84.03
HCT-116	78.99
HCT-15	80.09
HT29	72.08
KM12	80.41
SW-620	87.09
CNS Cancer	
SF-268	55.47
SF-295	85.38
SF-539	82.55
SNB-19	68.77
SNB-75	-14.55
U251	85.67
Melanoma	
LOX IMVI	93.99
MALME-3M	84.12
M14	87.24
MDA-MB-435	85.86
SK-MEL-2	20.42
SK-MEL-28	73.65
SK-MEL-5	62.17
UACC-257	74.36
UACC-62	52.43
Ovarian Cancer	
IGROV1	81.75
OVCAR-3	63.53
OVCAR-4	40.22
OVCAR-8	45.70
NCI/ADR-RES	71.07
SK-OV-3	69.52
Renal Cancer	
786-0	83.97
A498	66.42
ACHN	71.37
CAKI-1	84.91
RXF 393	43.34
SN12C	84.31
TK-10	52.96
Prostate Cancer	
PC-3	67.53
DU-145	85.25
Breast Cancer	
MCF7	65.30
HS 578T	31.71
BT-549	78.86
MDA-MB-468	71.43
Mean	67.35
Delta	81.90
Range	108.54

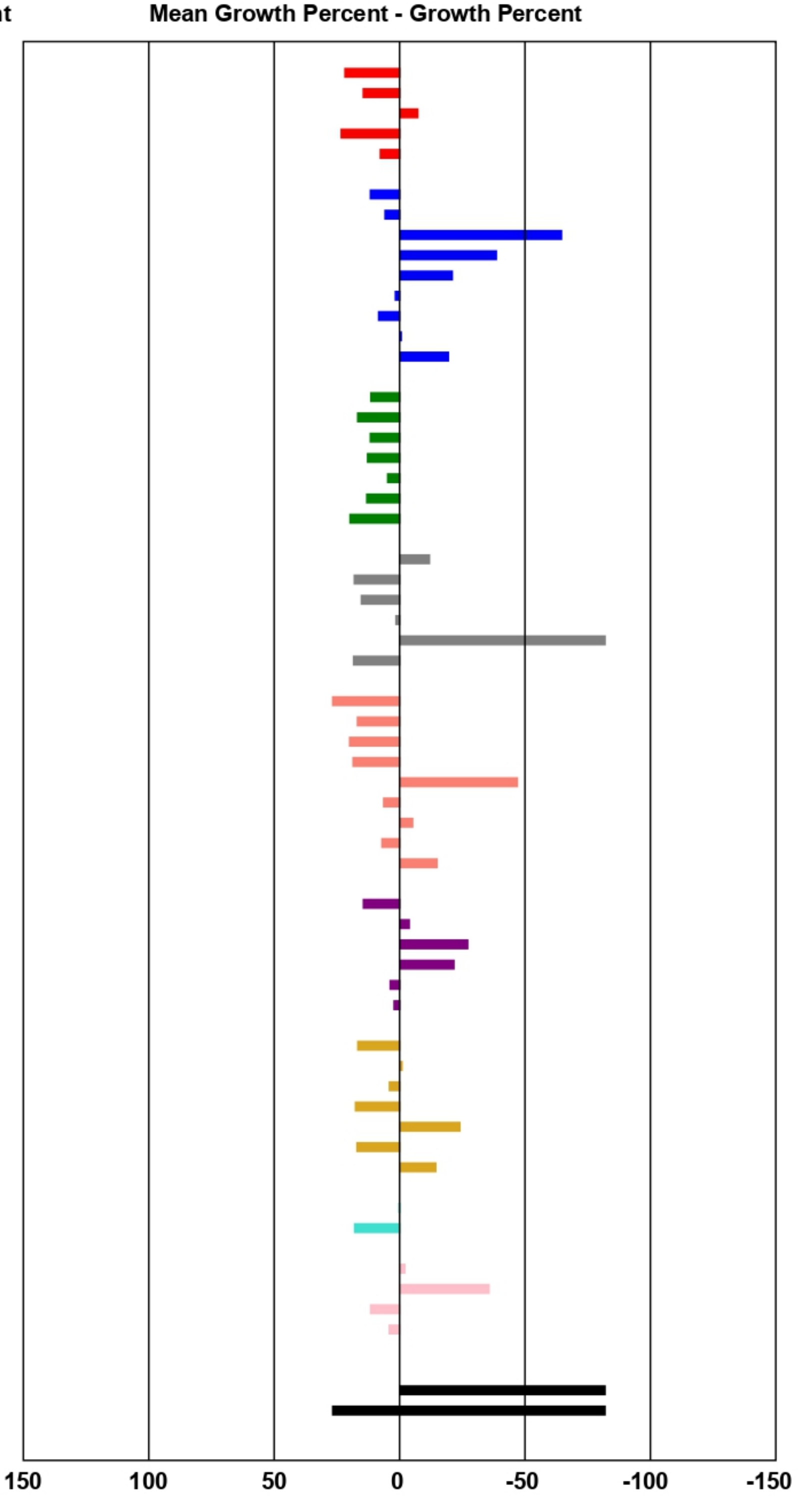

Figure S19. Results of the *in vitro* growth of cancer cell lines in the single-dose assay for compound **6c**

Developmental Therapeutics Program One Dose Mean Graph	NSC: D-850648 / 1	Conc: 1.000E-5 M	Test Date: Jan 8, 2024
	Experiment ID: 2401HT23		Report Date: Feb 22, 2024

Panel/Cell Line	Growth Percent
Leukemia	
CCRF-CEM	76.00
HL-60(TB)	51.28
K-562	110.08
MOLT-4	45.97
RPMI-8226	89.26
Non-Small Cell Lung Cancer	
A549/ATCC	94.40
EKVX	93.98
HOP-62	79.48
HOP-92	94.26
NCI-H226	91.96
NCI-H23	90.62
NCI-H322M	98.95
NCI-H460	97.38
NCI-H522	45.77
Colon Cancer	
COLO 205	124.45
HCC-2998	108.58
HCT-116	96.44
HCT-15	72.91
HT29	99.86
KM12	94.12
SW-620	83.29
CNS Cancer	
SF-268	104.05
SF-295	84.22
SF-539	99.27
SNB-19	90.52
SNB-75	88.00
U251	96.11
Melanoma	
LOX IMVI	93.01
MALME-3M	96.67
M14	114.59
MDA-MB-435	114.15
SK-MEL-2	94.47
SK-MEL-28	105.65
SK-MEL-5	41.09
UACC-257	94.42
UACC-62	101.10
Ovarian Cancer	
IGROV1	110.04
OVCAR-3	67.45
OVCAR-4	98.89
OVCAR-8	94.37
NCI/ADR-RES	108.33
SK-OV-3	90.72
Renal Cancer	
786-0	90.10
A498	98.17
ACHN	94.32
CAKI-1	97.92
RXF 393	90.60
SN12C	95.09
TK-10	90.50
Prostate Cancer	
PC-3	105.86
DU-145	93.42
Breast Cancer	
MCF7	87.05
HS 578T	90.25
BT-549	84.13
MDA-MB-468	72.82
Mean	91.21
Delta	50.12
Range	83.36

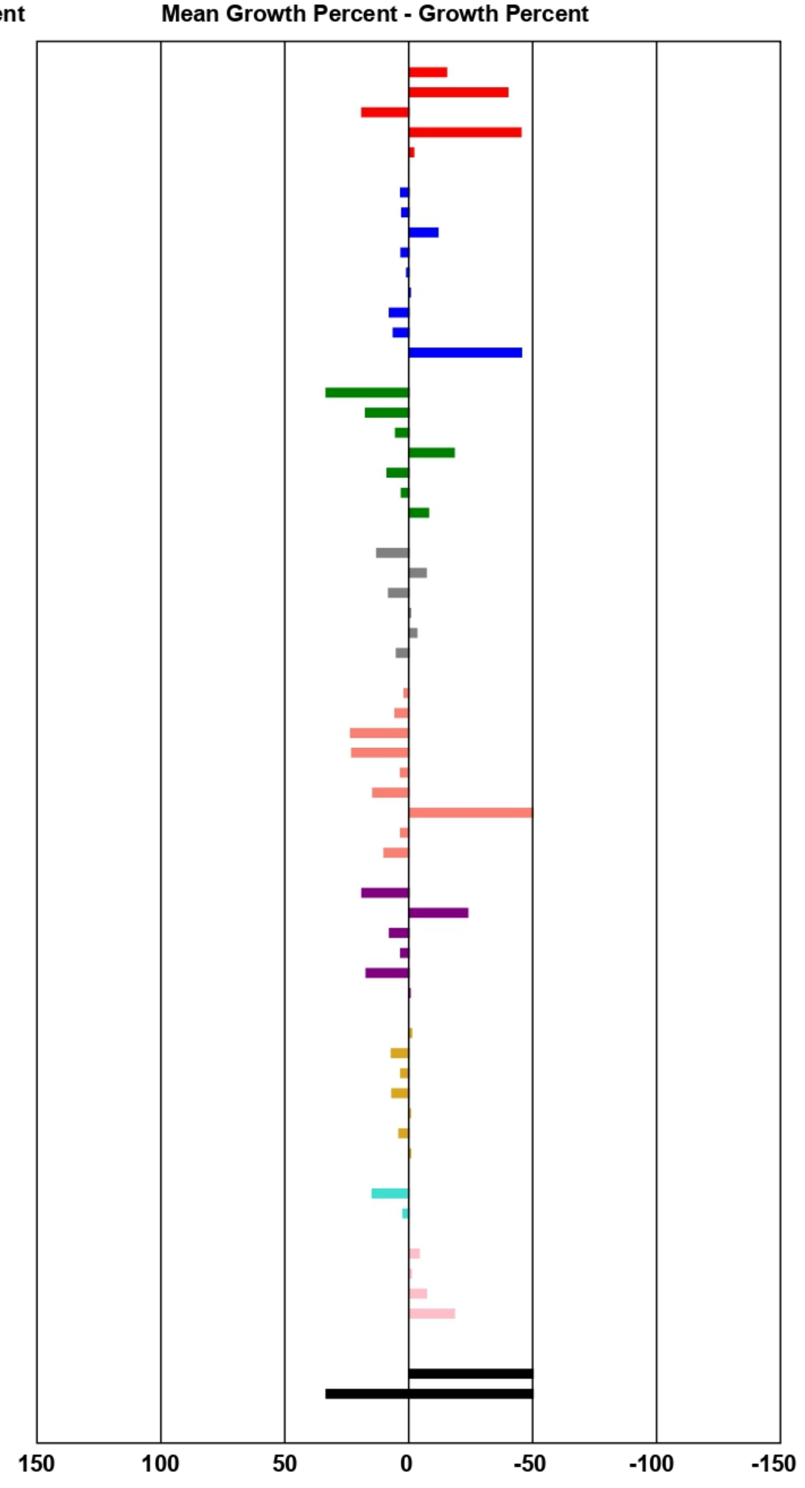

Figure S20. Results of the *in vitro* growth of cancer cell lines in the single-dose assay for compound **7d**

Developmental Therapeutics Program One Dose Mean Graph	NSC: D-850714 / 1	Conc: 1.000E-5 M	Test Date: Jan 8, 2024
	Experiment ID: 2401HT23		Report Date: Feb 22, 2024

Panel/Cell Line	Growth Percent
**Leukemia**	
CCRF-CEM	86.85
HL-60(TB)	64.10
K-562	103.72
MOLT-4	63.57
RPMI-8226	109.80
**Non-Small Cell Lung Cancer**	
A549/ATCC	84.03
EKVX	81.54
HOP-62	-15.20
HOP-92	65.65
NCI-H226	53.61
NCI-H23	49.93
NCI-H322M	68.93
NCI-H460	95.77
NCI-H522	56.61
**Colon Cancer**	
COLO 205	84.39
HCC-2998	86.69
HCT-116	58.99
HCT-15	57.86
HT29	87.83
KM12	68.20
SW-620	64.39
**CNS Cancer**	
SF-268	74.93
SF-295	98.93
SF-539	84.66
SNB-19	65.19
SNB-75	36.02
U251	70.41
**Melanoma**	
LOX IMVI	99.71
MALME-3M	63.85
M14	101.76
MDA-MB-435	93.06
SK-MEL-2	85.44
SK-MEL-28	68.24
SK-MEL-5	31.55
UACC-257	96.93
UACC-62	88.65
**Ovarian Cancer**	
IGROV1	85.67
OVCAR-3	45.60
OVCAR-4	79.52
OVCAR-8	83.83
NCI/ADR-RES	88.75
SK-OV-3	104.17
**Renal Cancer**	
786-0	100.09
A498	83.86
ACHN	91.25
CAKI-1	90.65
RXF 393	91.62
SN12C	91.12
TK-10	90.17
**Prostate Cancer**	
PC-3	91.91
DU-145	98.34
**Breast Cancer**	
MCF7	37.85
HS 578T	88.79
BT-549	71.62
MDA-MB-468	102.58
Mean	77.34
Delta	92.54
Range	125.00

Mean Growth Percent - Growth Percent

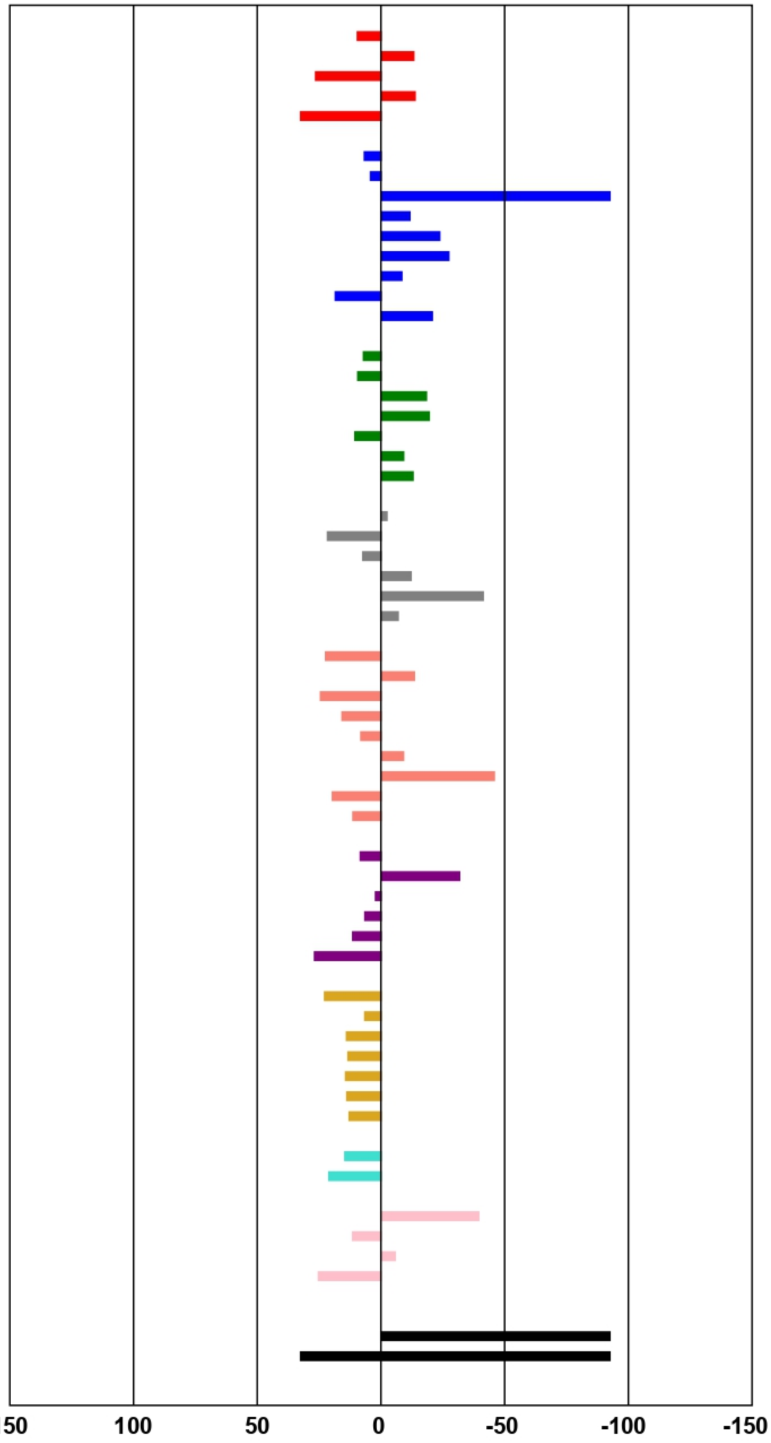

Figure S21. Results of the *in vitro* growth of cancer cell lines in the single-dose assay for compound **7g**

Table S1. Crystal data and details of structure refinement for compound **6a**

	**6a**
Emp. formula	$C_{18}H_{13}BrClNO_3$
Fw	406.65
T [K]	293(2)
space group	P-1
a [Å]	8.8163(6)
b [Å]	9.1251(7)
c [Å]	11.6421(9)
α [°]	77.035(7)
β [°]	72.147(7)
γ [°]	69.349(7)
V [Å^3]	827.08(12)
Z	2
ρ_{calcd} [g cm^{-3}]	1.633
μ [mm^{-1}]	2.662
Crystal size [mm]	0.25×0.2×0.2
2Θ range	3.708 to 50.046
Refls. collected	7028
Indep. Refls., R_{int}	2856, 0.0402
Data/rests./params.	2856/0/219
GOF	1.023
R_1, wR_2(all data)	0.0346, 0.0747
CCDC no.	2477072

Table S2. Bond distances (Å) and angles (°) for **6a**

Br1-C2	1.881(3)	C4-C5	1.341(4)
Cl1-C16	1.744(3)	C6-C7	1.378(4)
O1-C9	1.352(3)	C6-C12	1.452(4)
O1-C10	1.451(3)	C7-C8	1.383(4)
O2-C9	1.198(3)	C8-C9	1.465(4)
O3-C12	1.224(3)	C10-C11	1.444(5)
N1-C1	1.399(3)	C12-C13	1.497(4)
N1-C5	1.376(3)	C13-C14	1.383(4)
N1-C6	1.394(4)	C13-C18	1.395(4)
C1-C2	1.409(4)	C14-C15	1.374(4)
C1-C8	1.421(3)	C15-C16	1.371(4)
C2-C3	1.364(4)	C16-C17	1.366(4)
C3-C4	1.394(4)	C17-C18	1.376(4)

C9-O1-C10	117.1(2)	C7-C8-C9	122.8(3)
C5-N1-C1	122.8(3)	O1-C9-C8	110.2(2)
C5-N1-C6	127.2(3)	O2-C9-O1	122.9(2)
C6-N1-C1	109.9(2)	O2-C9-C8	126.8(3)
N1-C1-C2	116.7(2)	C11-C10-O1	109.8(3)
N1-C1-C8	106.0(2)	O3-C12-C6	123.3(3)
C2-C1-C8	137.2(3)	O3-C12-C13	120.6(3)
C1-C2-Br1	121.8(2)	C6-C12-C13	116.0(2)
C3-C2-Br1	118.0(2)	C14-C13-C12	119.5(3)

C3-C2-C1	120.0(3)	C14-C13-C18	118.4(3)
C2-C3-C4	120.6(3)	C18-C13-C12	122.0(3)
C5-C4-C3	121.0(3)	C15-C14-C13	120.7(3)
C4-C5-N1	118.8(3)	C16-C15-C14	119.5(3)
N1-C6-C12	124.4(2)	C15-C16-Cl1	119.0(3)
C7-C6-N1	106.3(2)	C17-C16-Cl1	119.5(2)
C7-C6-C12	128.8(3)	C17-C16-C15	121.4(3)
C6-C7-C8	110.5(3)	C16-C17-C18	119.0(3)
C1-C8-C9	128.6(3)	C17-C18-C13	120.9(3)
C7-C8-C1	107.2(2)		
